# Effect of hydrogen sulfide on alpha-synuclein aggregation and cell viability

**DOI:** 10.1038/s41598-025-99794-z

**Published:** 2025-05-04

**Authors:** Elena A. Ostrakhovitch, Eun-Suk Song, Johannah E. Stegemann, Michael McLeod, Tritia R. Yamasaki

**Affiliations:** 1 Department of Neurology, University of Kentucky, 740 South Limestone St., Ste. J401, Lexington, KY 40536-0284 USA; 2 Veterans Affairs, Lexington, KY 40536 USA

**Keywords:** α-Synuclein, Parkinson disease, Protein aggregation, Hydrogen sulfide

## Abstract

Parkinson’s disease (PD) is a progressive neurodegenerative movement disorder characterized by nigrostriatal degeneration and aggregation of α-synuclein (α-Syn) with accumulation of insoluble aggregates in Lewy bodies. Familial mutations in α-Syn are associated with the development of PD. Accumulation of insoluble aggregates results in neuronal toxicity. Identification of compounds that inhibit seeding activity of α-Syn is of great importance. Here we investigate the potential of H2S donor, sodium hydrosulfide (NaHS), to inhibit α-Syn aggregation. We examined the effect of NaHS on fibril growth kinetics and the structural change of α-Syn fibrils formed by self-seeding and cross-seeding of wild-type (wt) and PD familial α-Syn mutations. NaHS slowed both self- and cross-seeded A53T α-Syn fibril formation but not wild-type fibril formation. We observed a decrease in the formed fibril length in vitro. We examined the effect on fibril formation within cells. NaHS significantly reduced the number and filament length of formed oligomers in an α-Syn overexpressing cell model. Furthermore, NaHS rescued viability of A53T α-Syn overexpressing cells seeded with wt- and mutant preformed fibrils. These results support a conformation-specific effect of hydrogen sulfide on alpha-synuclein aggregation and cell viability which deserves further exploration for therapeutic potential.

**Supplementary Information:**

The online version contains supplementary material available at 10.1038/s41598-025-99794-z.

## Introduction

Parkinson disease is a progressive degenerative neurologic movement disorder predominantly affecting dopaminergic neurons^1,2^. It affects nearly 1% of adults over 60 years of age worldwide. The prevalence of PD is increasing over the years as the population is aging rapidly worldwide^3–5^. Alpha-synuclein (α-Syn) is a 14 kDa protein that is linked neuropathologically to the majority of cases of Parkinson’s disease (PD). It localizes to the synapses and nuclear envelope. α-Syn exists predominantly as a disordered monomeric protein and adopts helical conformation in the membrane bound state^6,7^. α-Syn is characterized by significant conformational plasticity and pathologic prion-like properties^6^. Under pathological conditions, α-Syn forms oligomeric amorphous aggregates and fibrillar insoluble aggregates that have the ability to amplify by recruiting and promoting misfolding of the endogenous α-Syn resulting in accumulation into Lewy body (LB) inclusions^8^.

Single point mutations associated with familial Parkinson’s disease affect α-Syn conformation and aggregation propensity. A53T and H50Q mutations accelerate α-Syn fibrillization^9–11^. A53T mutation enhances α-Syn aggregation by reducing the unfavorable activation enthalpy of nucleation^12^. G51D mutation in α-Syn causes impairment of protein membrane association leading to the formation of non- toxic inclusions^9^. A newly described A30G mutation alters the protein’s α-helical structure and membrane binding^13^. Despite several studies, the basis for the differences in the fibrillization/oligomerization properties of wild-type (wt) α-Syn and mutants remains unclear.

Numerous studies have been conducted to understand the mechanisms underlying the enhanced aggregation and fibril formation with the goal of developing therapies to combat PD-associated α-Syn oligomerization and fibrillization. Several studies have shown that polyphenols exert antioxidant action and suppress the formation of α-Syn fibrils by interfering with β-sheet structure formation^14–16^. It was suggested that small molecules that reduce the aggregation rate or affect oligomer conformation interfere with α-Syn fibrillization by redirecting assembly into non-amyloidogenic structures^17^.

Hydrogen sulfide (H2S) is a main gasotransmitter, in addition to nitric oxide (NO) and carbon monoxide (CO). H2S is endogenously generated through the transfer of sulfur in the transsulfuration pathway by cystathionine γ-lyase (CSE) and cystathionine β-synthase (CBS)^18,19^. It is also produced from 3- mercaptopyruvate (3MP) by mitochondrial 3-mercaptopyruvate sulfurtransferase (3-MST) and cysteine/GSH disulfide conjugates by mitochondrial thiosulfate-cyanide sulfurtransferase (TST). TST activity modulates mitochondrial complex activity. It reduces nicotinamide adenine dinucleotide (NADH) dehydrogenase (Complex I) and succinate dehydrogenase (Complex II) via interaction with their iron- sulfur clusters^20^. H2S inhibits generation of free radicals and protects brain cells from mitochondrial, Golgi, lysosomal, and endoplasmic reticulum stress^21–24^. Prior studies demonstrate that H2S exerts neuroprotective effects in various neurodegenerative conditions^25,26^. It was reported that H_2_S reduces the production of β-secretase and presenilin, the enzymes that process amyloid precursor protein into β-amyloid fragments^27^. In in vivo models of Parkinson’s disease, administration of an H_2_S donor reduced oxidative stress and attenuated the loss of dopaminergic neurons^28,29^.

With evidence that H2S reduces oxidative damage and has anti-inflammatory and anti-amyloid properties, we tested the ability of hydrogen sulfide to regulate alpha-synuclein oligomerization/fibrillization utilizing H2S donor molecule sodium hydrosulfide (NaHS). NaHS in aqueous solution results in release of H2S^30^. We examined the effect of NaHS on nucleation and elongation of wild-type (wt) and familial PD forms of α- Syn. Using transmission electron microscopy (TEM), we visualized the structural difference in fibrils following exposure to H2S. Finally, we tested the ability of H2S to inhibit the formation of intracellular aggregates in α-Syn overexpressing cells. We find that NaHS inhibits fibril formation and attenuated the cytotoxicity associated with fibrils in a mutation-specific manner.

## Results

### Effect of familial PD mutations on spontaneous and seeded aggregation

We first examined the fibril formation utilizing a seed amplification assay. We performed Endpoint Quaking-induced conversion (EP-QuIC)^31,32^. Similar to real time quaking induced conversion (RT-QuIC), this assay is based on the detection of amyloid fibril formation using a thioflavin T (ThT) fluorescence readout. EP-QuIC shortens the response time and allows the use of any plate reader with the correct detection and emission parameters. Most RT-QuIC protocols utilize double orbital shaking, whereas in the EP-QuIC method we utilized circular shaking.

**Fig. 1:**
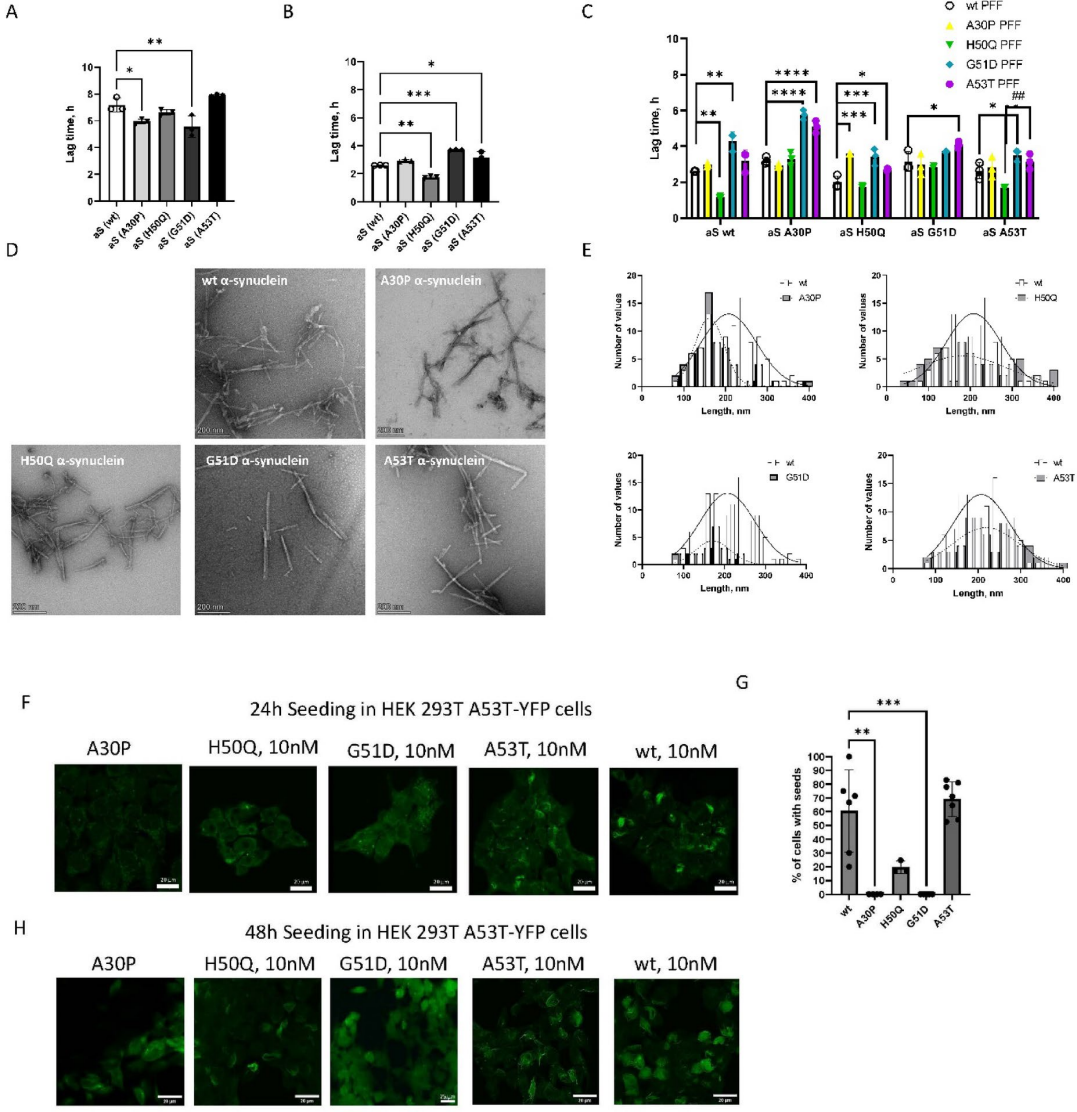
α-Syn familial mutation effects on fibril amplification in vitro and aggregation within cells. (**A**–**C**) Fibril formation kinetics of human α-Syn monitored by ThT. (**A**) Comparison of lag time for fibril growth of spontaneous (non-seeded) and (**B**) self-seeded aggregation of α-Syn monomer. (**C**) Comparison of lag time for fibril growth of cross-seeded aggregation of α-Syn monomer. Monomeric forms of recombinant wt-, A30P-, H50Q-, G51D- and A53T-α-Syn mutant were seeded with wt- and mutated α-Syn PFF. The results of ThT analysis represent the average of several experiments (at least n = 3, in some instances n = 5). Error bars represent S.E. *p < 0.05, **p < 0.005, ***p < 0.0005. ****p < 0.0001, ^##^*p* < 0.005. (**D**,**E**) Characterization of wt fibrillar α-Syn and fibrillar α-Syn bearing mutation at A30P, H50Q, G51D and A53T. (**D**) Representative TEM images of 1 µM wt α-Syn and α-Syn bearing mutation at A30P, H50Q, G51D and A53T fibrils. (**E**) α-Syn fiber length distribution. Fiber lengths for mutations are plotted against the same wt values for each graph. The figures are representative of three independent experiments with 3 grids analyzed per condition. (**F**–**H**) Analysis of propagation of internalized wt- and mutated α-Syn PFF in HEK 293 T cells overexpressing α-Syn with familial point mutation A53T tagged with YFP (HEK 293 T A53T-YFP). Cells were transduced with 10 nM A30P-, H50Q-, G51D-, A53T-, and wt-α-Syn seeds for 24 h and 48 h. Inclusions were validated with confocal microscopy (Nikon Eclipse Ti2). (**F**) Representative confocal images of seeded HEK 293 T A53T-YFP cells for 24 h (Scale bar, 20 µm). (**G**) Histogram of the percentage of cells with α-Syn inclusions 24 h following transduction with 10 nM α-Syn seeds. Statistical significance is shown as **p < 0.005, ****p < 0.0001. (**H**) Representative confocal images of seeded cells transduced with 10 nM A30P-, H50Q-, G51D-, A53T-, and wt-αSyn for 48 h. For evaluation, three independent staining experiments were analyzed.

The QuIC process is divided into initial lag phase, growth phase, and plateau phase. The lag phase represents the time required for seeds to contact the substrate and trigger conformational change. We utilized α-Syn monomer and pre-formed fibrils (PFF) with wt- and familial PD mutations (A30P, H50Q, G51D, and A53T) to determine the effect of primary structural differences on fibrillogenesis. We monitored spontaneous (non-seeded) fibril formation at pH 7.4 in 10 mM HEPES and 400 mM NaCl at 45 °C. Analysis of lag times for non-seeded α-Syn indicated the shorter lag time for A30P and G51D variants (Fig. 1A). However, there was no difference in lag times between wt, A53T, and H50Q. The protein aggregation rate (PAR), defined as an inverse of the lag phase, did not significantly differ between wt and mutants; however, the G51D and H50Q mutation accelerated the fibrilization of α-Syn (Suppl. Figure 1A-B) based on an increased PAR.

In accordance with a nucleation-dependent polymerization model of amyloid assembly, growth of fibrils is accelerated in the presence of seeds^33^. Since heterozygous α-Syn mutations exist in most patients with familial SCNA gene mutations and mutant α-Syn can induce wt monomer to form fibrils^34^, we next explored the changes induced when monomer was self-seeded with the same wt or mutation in the monomer and fibril seed (homotypic) or cross-seeded with different wt or mutation in the monomer and fibril seed (heterotypic). A comparison of lag times for homotypic reactions showed that wt and H50Q replicated significantly faster, whereas G51D and A53T replicated more slowly (Fig. 1B, Suppl. Figure 1C-D). We also monitored the fibril growth kinetics in the heterotypic reactions. The lag phase was significantly shortened when wt and A53T α-Syn were cross-seeded with H50Q variant PFF (Fig. 1C, Suppl. Figure 1E-I). Interestingly, A53T, G51D, and, in some instances, A30P cross-seeding slowed down fibril formation (Fig. 1C).

To directly visualize the effect of self-seeded aggregation on fibril structure, negative-stain TEM images were examined (Fig. 1D). Different polymorphs were observed for different mutations. Rod-shaped fibrils were observed in wt, H50Q and A53T α-Syn mutants. The A30P mutation was associated with the formation of branched, twisted, and laterally associated fibrils. G51D fibrils were shorter and more fragmented. H50Q and A53T fibrils had the highest distribution between 140–320 nm in length (Fig. 1E) and 9–20 nm in width (Suppl. Figure 2A-B) whereas wt fibrils measured up to 400 nm in length, yet were of similar width. A30P and G51D α-Syn fibrils were considerably shorter and measured 110–220 nm in length and 8–12 nm in width. Additional micrographs are shown in Suppl. Figure 4.

We next used HEK 293 T cells overexpressing A53T α-Syn-YFP (as generated and utilized^35^) to evaluate seeding activity of amplified fibrils in a cellular biosensor described previously^35^. The seeding behavior of PFFs was dependent on the type of mutation. The seed competence of A30P and G51D α-Syn PFFs was very low. Cells seeded with wt and A53T α-Syn PFFs contained long filamentous strands spreading throughout the cytoplasm (Fig. 1F-G, Suppl. Figure 3A). Although H50Q α-Syn PFFs strongly amplified A53T fibril formation in the QuIC assay, they were much less efficient in seeding in a cell-based system compared to wt and A53T α-Syn PFF. Aggregates induced by H50Q had a more punctate morphology at the early (24 h) timepoint but developed into more filamentous inclusions by 72 h (Suppl. Figure 3A).

Similar trends toward smaller inclusion size in A30P, H50Q, and G51D were observed in HEK 293 T overexpressing wt α-Syn-YFP (Suppl. Figure 3B-C). However, seeding activity of H50Q and G51D PFFs was higher and morphology was more punctate. The diverse morphology of inclusions seen in α-Syn wt and A53T mutant overexpressing cells also suggests different structural assembly of the underlying intracellular aggregates.

### Effect of sodium hydrosulfide (NaHS) on α-Syn aggregation kinetics

**Fig. 2:**
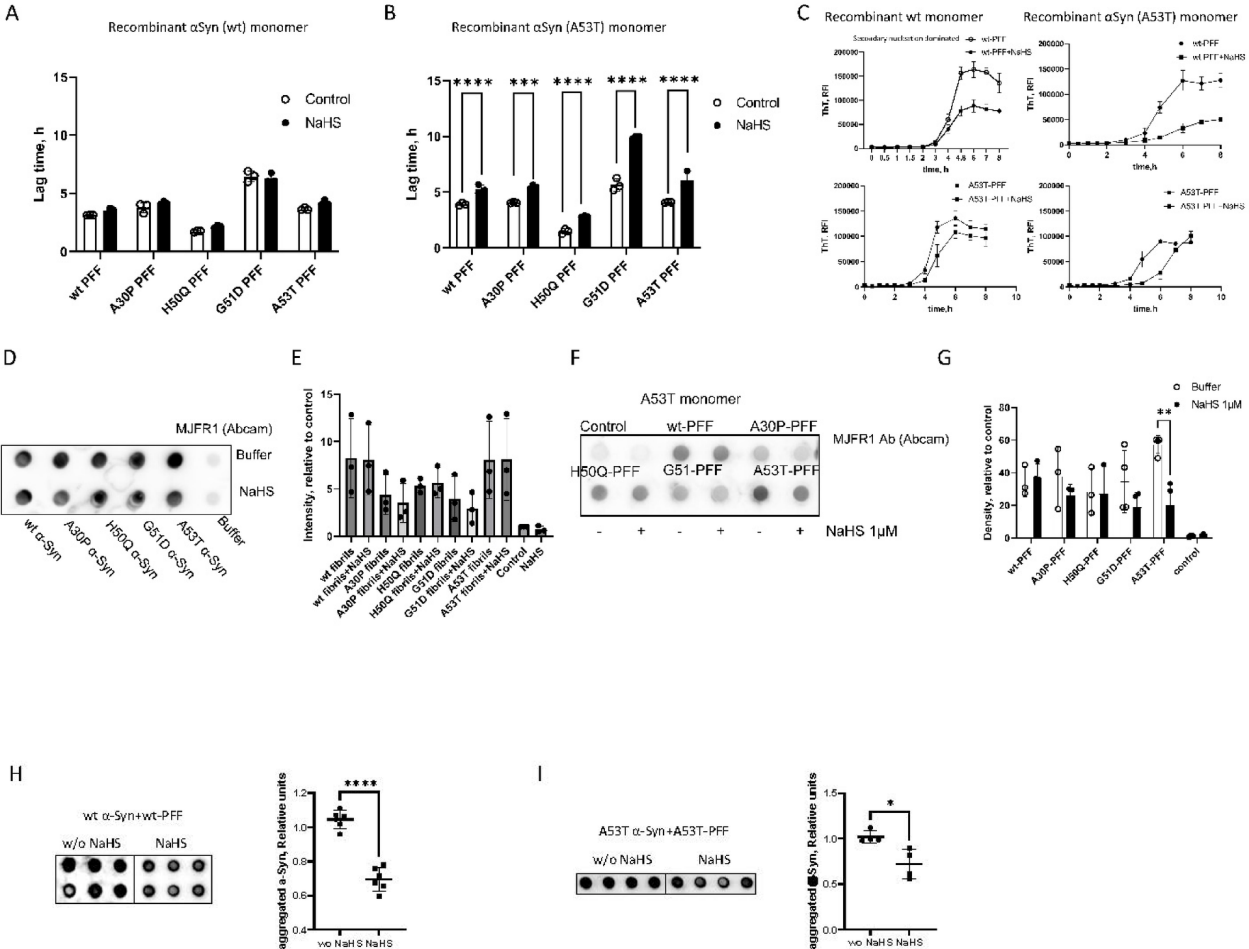
NaHS interferes with A53T aggregation in self- and cross-seeding paradigms by prolonging the lag phase and delaying fibril formation. (**A**) Comparison of lag time for fibril growth of wt-α-Syn monomer seeded with wt PFF and cross-seeded with α-Syn variants (A30P, H50Q, G51D and A53T) in the absence and presence of 1µM NaHS. (**B**) Comparison of lag time for fibril growth of A53T-α-Syn monomer self-seeded and cross-seeded with wt and α-Syn variants (A30P, H50Q, G51D) in the absence and presence of 1µM NaHS. Error bars represent. S.E. ***p < 0.0005. **** *p* < 0.0001. (**C**) ThT fluorescence traces for fibril formation of A53T α-Syn monomer seeded with wt PFF and A53T PFF in the absence and presence of 1 µM NaHS. The curve is the average of 3 independent experiments. (**D**) Representative dot blot of α-Syn in non-seeded QuIC samples in the absence and presence of NaHS (n = 3). α-Syn was detected using anti-α-Syn MJFR1 Ab. (**E**) Quantification of dot blot of αSyn in seeded QuIC samples in the absence and presence of 1µM NaHS (n = 2). α-Syn was detected using mouse anti-αSyn Ab clone 42 from BD Biosciences. (**F**) Representative Dot blot of α-Syn in PFF-seeded QuIC samples in the absence and presence of 1µM NaHS (n = 3). Control is non-seeded QuIC samples. α-Syn was detected using anti-α-Syn MJFR1 Ab. (**G**) Quantification of dot blot, each dot represents an individual sample. presence of 1 µM NaHS. (**H**,**I**) Representative dot blot and histogram analysis of wt- (n = 6) (**H**), and A53T α-Syn (n = 4) (**I**) in A53T-PFF-seeded QuIC samples in the absence and presence of 1µM NaHS. α-Syn was detected using anti-Alpha-synuclein aggregate antibody [MJFR-14–6-4–2]. Error bars represent. S.E. *p < 0.05. ****p < 0.0001.

To test the effect of NaHS, the most widely used H2S donor, on α-Syn fibrillization, we set up series of in- plate wt and A53T α-Syn aggregation reactions initiated by nucleation with wt and mutant α-Syn seeds. Previously, it has been shown that NaHS does not exhibit toxicity at concentrations of up to 400 µM in HEK-293 and SH-SY5Y cell cultures in short-term studies (30 min)^29^. However, long term exposure to high concentrations of H2S suppresses cellular respiration and leads to cell death. The activity of mitochondrial cytochrome c oxidase is abolished by a solution of NaHS at concentrations ranging from 10 to 30 µM^36^. Based on this evidence, we tested the effect of 1 µM NaHS . NaHS did not change ThT fluorescence signal as assessed over a range of excitation and emission spectra (Suppl. Figure 5A). The presence of 1 µM NaHS did not lead to any significant changes in dynamics of wt α-Syn fibril elongation initiated by wt or mutant α-Syn seeds (Fig. 2A). However, NaHS significantly prolonged the lag time, and thus slowed A53T α-Syn fibril formation initiated by wt and mutant α-Syn seeds (Fig. 2B,C; Suppl. Figure 5B). These results indicate that A53T monomer aggregation is prominently inhibited by H2S regardless of seeding PFF structure. The decrease in fibril formation is evident by analysis of fibrilization products. Immunoblot (dot blot) analysis with MJFR1 α-synuclein antibody (Abcam) indicates that the presence of NaHS did not change the level of α-Syn in spontaneous aggregation (Fig. 2D-E). Supporting the finding that H2S maintains A53T in the monomeric state, ThT fluorescence traces (Fig. 2C) indicate that NaHS significantly decreased the number of A53T formed fibrils initiated by wt α-Syn seeds as shown by the maximal fluorescence at the plateau phase of the reaction. We analyzed our data (Fig. 2C) using AmyloFit 2.0 (https://amylofit.com/amylofitmain/fitter/)^37^. Fitting the reaction into the secondary nucleation model allowed us to estimate the K_+_, which denotes the aggregation rate constants for growth in the inhibitor’s absence and presence with a mean residual error of less than 0.002. For recombinant α-Syn A53T monomer seeded with wt-PFF: K_+_(“-NaHS”) = 5.09e^+9^ K_+_(“ + NaHS”) = 1.2e^+9^; and A53T-PFF: K_+_(“-NaHS”) = 3.8e^+9^ ; A53T-PFF: K_+_(“ + NaHS”) = 1.08e^+9^. For recombinant α-Syn wt monomer seeded with A53T-PFF: K_+_(“-NaHS”) = 5.25e^+9^ ; A53T-PFF: K_+_(“ + NaHS”) = 1.44e^+9^. Dot-blot analysis shows a decrease in A53T α-Syn post-amplification with A53T-PFFs but not other types of PFFs (Fig. 2F–I). These results further support our finding that H2S affects A53T fibrillization.

**Fig. 3:**
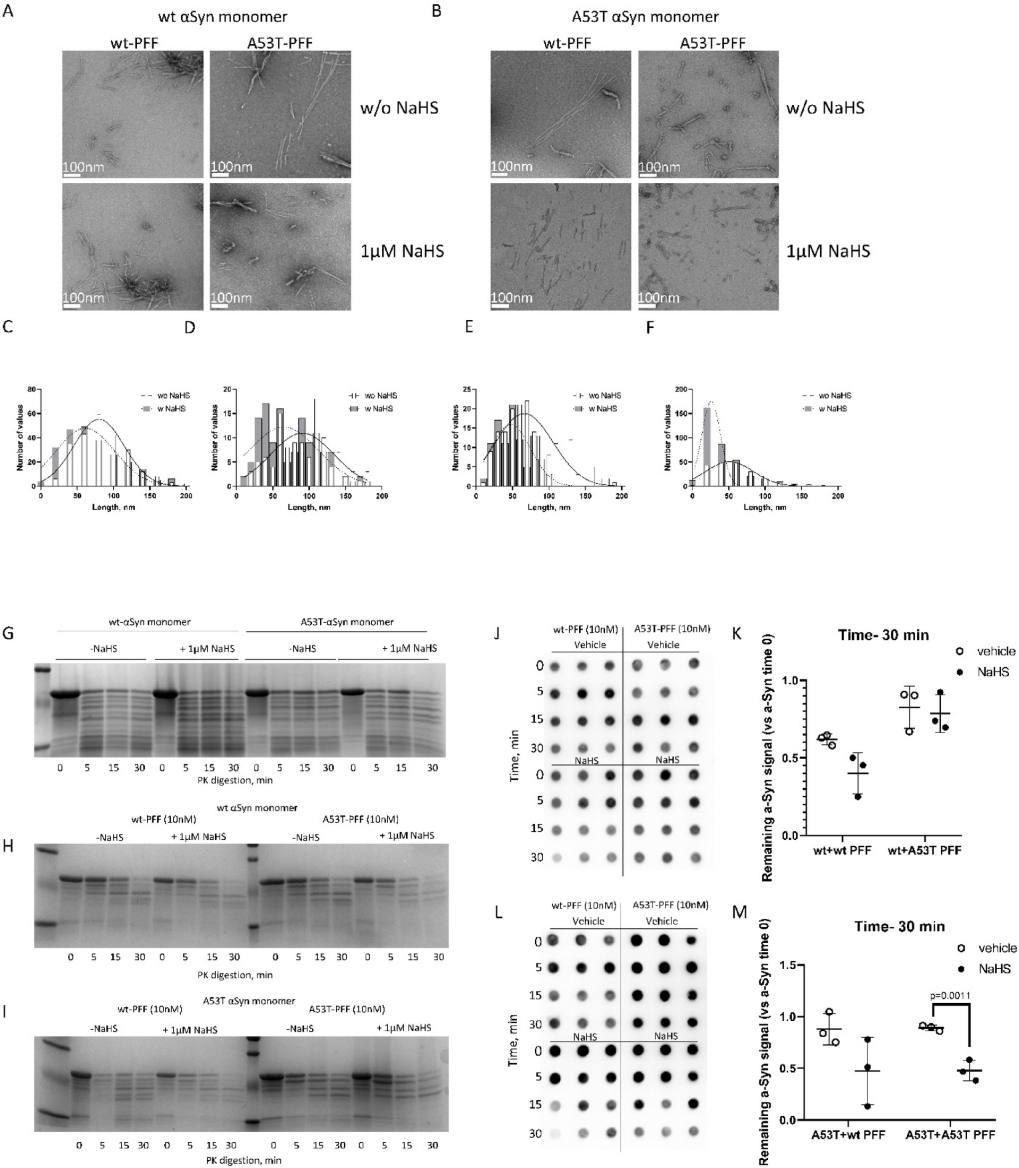
Treatment with 1µM NaHS reduced mature fibril length. (**A**,**B**) Uranyl acetate-stained TEM micrographs of α-Syn fibrils generated by 3 days of shaking in the presence of NaHS. The wt-PFF and A53T-PFF acted as seeds to induce wt α-Syn (**A**) and A53T α-Syn (**B**) fibrilization in the absence and the presence of 1 µM NaHS. (**C**–**D**) Fibril length distribution for wild-type α-Syn monomer seeded with either (**C**) wt-PFF or (**D**) A53T- PFF. (**E**–**F**) Fibril length distribution for A53T α-Syn seeded with either (**E**) wt-PFF or (**F**) A53T-PFF. The figures are representative of three independent experiments with 3 grids analyzed per condition. (**G**–**I**) Stability of α-Syn fibrils against proteolysis. Aggregation was carried out under QuIC conditions. PK digestion was performed at 37 °C at the indicated period. (**G**) Proteinase K (PK) resistance of wt- and A53T α-Syn fibers which were replicated in the absence of PFF seeding show no difference in PK resistance in the absence or the presence of 1 µM NaHS. (**H**) PK resistance of wt α-Syn fibrils formed by seeding with wt- and A53T-PFF in the absence and presence of 1 µM NaHS. (**I**) PK sensitivity of wt and A53T α-Syn fibrils produced by seeding with PFF under ThT conditions in the absence and presence of 1 µM NaHS. (**J**) α-Syn dot-blot immunostaining after proteinase K (PK)-digestion of wt α-Syn fibrils formed by seeding with wt- and A53T-PFF in the absence and presence of 1 µM NaHS. (**K**) Quantification of dot-blot, each dot represents an individual sample. (**L**) α-Syn dot-blot immunostaining after proteinase K (PK)-digestion of A53T α-Syn fibrils formed by seeding with wt- and A53T-PFF in the absence and presence of 1 µM NaHS. (**M**) Quantification of dot-blot, each dot represents an individual sample. presence of 1 µM NaHS. (**M**) Quantification of dot-blot, each dot represents an individual sample.

Negative TEM staining was performed to examine fibril structure following treatment with NaHS (Fig. 3A–F). NaHS slightly reduced the length of wt α-Syn fibrils (Fig. 3A,C,D). However, there was a more striking effect of NaHS treatment on A53T α-Syn monomer seeded with PFFs. NaHS greatly reduced fibril length when A53T α-Syn was seeded with either wt or A53T PFFs (Fig. 3B,E, Suppl. Figure 4). When seeded with wt-PFFs, the mature fibrils appeared straight and showed a mean length of 80 ± 40 nm. In the presence of NaHS, fibril length decreased to a mean of 40 ± 30 nm. When seeded with A53T-PFFs, the mature fibrils appeared twisted and showed a mean length of 50 ± 30 nm. NaHS decreased fibril length to 30 ± 20 nm (Fig. 3B,F).

Next, we studied whether non-seeding (spontaneous aggregation) and seeding of wt and A53T mutant α- Syn with different PFFs in the presence of NaHS would result in species with different conformational properties as determined by proteinase K (PK) digestion (Fig. 3G–I). Consistent with the lack of the effect of NaHS during non-seeded (spontaneous) fibrilization during QuIC amplification (Fig. 2A), the fragmentation profile from a 30 min PK digestion for non-seeded samples revealed similar bands between wt and A53T conditions (Fig. 3G) implying that NaHS did not alter the properties of the resultant fibrils. The fragmentation profile from a 90 min PK digestion revealed that NaHS decreased fibrils resistance to PK (Suppl. Figure 6C). We did not see much change in PK sensitivity in NaHS pre-treated wt monomeric samples seeded with either wt or A53T PFFs (Fig. 3H–I, full-length gel images in Suppl. Figure 8). In the Western-blot and dot-blot immunoassay, NaHS decreased A53T resistance to PK digestion as evidenced by the remaining ɑ-syn signal (Fig. 3L,M, Suppl. Figure 6A,B,E). NaHS pre-treatment of A53T monomeric solution upon seeding with A53T PFF resulted in formation of fibrils with a higher PK sensitivity (Fig. 3H,M, Suppl. Figure 6A,B,E).

**Fig. 4:**
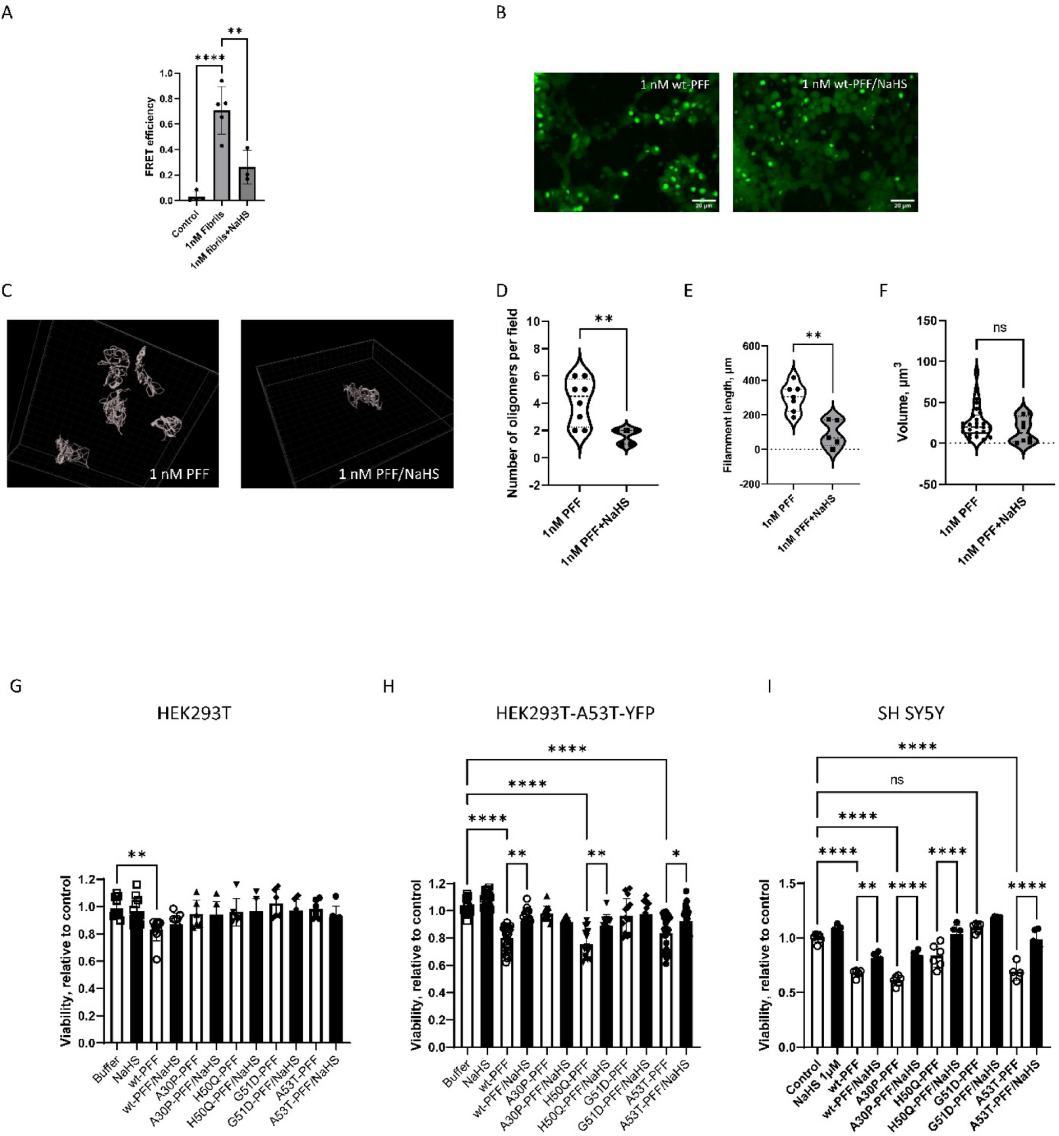
NaHS diminished intracellular seeding activity, size and cytotoxicity of α-Syn PFFs. (**A**) FRET efficiency values for HEK 293T A53T CFP/YFP as a measure of α-Syn aggregation. (**B**) Representative confocal images of HEK 293T A53T-YFP cells seeded with wt-PFF and treated with 1 µM NaHS. The seeding was validated with confocal microscopy (Nikon Eclipse Ti2). The cells were transduced with 1 nM wt-α-Syn seeds for 48 h. Statistical significance is shown as **p < 0.005, ****p < 0.0001. (**C**–**F**) Visualization and analysis of oligomers (N-SIM, Nikon, with 100 × magnification) using Imaris (Bitplane) image analysis software. (**C**) Representative reconstructed 3D images of α-Syn aggregates formed in HEK293 A53T-YFP cells seeded with 1nM wt-PFF in the absence and the presence of 1 µM NaHS. (**D**) Number of aggregates per field. (**E**) Analysis of filament length. (**F**) Filament surface. Statistical significance is shown as *p < 0.05, **p < 0.005. (**G**–**I**) NaHS suppresses α-Syn toxicity in HEK 293T (**G**), HEK 293T cells overexpressing A53T mutant (**H**), and SH-SY5Y (**I**) seeded with preformed fibrils. Cells were treated with either buffer or 1μM NaHS. Cytotoxicity was assessed by XTT assay after exposure to 10nM α-Syn wt and 10nM α-syn mutated at A30P, H50Q, G51D and A53T fibrils for 3 days. Statistical significance of 5 independent experiments is shown as *p < 0.05, **p < 0.005, ***p < 0.0005. ****p < 0.0001.

Next, we examined inhibitory potential of 1 µM NaHS in HEK 293 T-A53T cells labeled with CFP and YFP. Fluorescence resonance energy transfer (FRET) signal detection allows assessment of the interaction between CFP and YFP-tagged monomer^38^. Cells were pretreated with NaHS and seeded with 1 nM α-Syn PFFs for 48 h. After the incubation, aggregates of fibrillized α-Syn were visualized using confocal microscopy (Nikon) and FRET was assessed using a FRET Analyzer plugin for ImageJ program (https://imagej.net/ij/plugins/fret-analyzer/fret-analyzer.html) that allows pixel-by-pixel analysis of FRET (Fig. 4A). The measurement of FRET showed that seeding with wt PFFs led to an eightfold increase of FRET efficiency compared to control without PFFs (Fig. 4A). Treatment with NaHS significantly decreased the FRET signal (Fig. 4A). Since the FRET signal is proportional to the amount of the formed aggregates, the data support intracellular NaHS suppression of α-Syn aggregation.

HEK 293 T-A53T cells labeled with YFP were pretreated with NaHS and seeded with 1 nM α-Syn PFFs for 48 h. The resultant aggregate formation was visualized using Nikon AX-R confocal microscope (Fig. 4B). Formed aggregates were then examined using N-SIM S Super-Resolution Microscopy (Nikon). The images were analyzed (Imaris, Oxford Instruments) and underwent 3D reconstruction for surface rendering and filament measurement (Fig. 4C). NaHS significantly reduced the number of aggregates per field (Fig. 4D) and significantly decreased filament length (Fig. 4E), but did not significantly change aggregate volume (Fig. 4F). GYY4137 (morpholin-4-ium 4-methoxyphenyl(morpholino) phosphinodithioate), a slow releasing H2S donor, at concentrations of 100 µM also decreased the number of formed aggregates (Suppl. Figure 6A,B).

Finally, we investigated the ability of NaHS to attenuate PFF-induced cell toxicity. HEK 293 T, HEK 293 T cells overexpressing A53T α-Syn, and SH-SY5Y were seeded with wt and mutant PFFs. HEK 293 T showed minimal sensitivity to PFFs, with only wt PFFs causing some decrease in viability (Fig. 4G–I). Exposure of A53T α-Syn overexpressing cells and SH-SY5Y cells to PFFs led to a decreased cell viability. The most prominent effect was observed when A53T overexpressing cells were seeded with wt, H50Q, and A53T PFFs (Fig. 4H). NaHS rescued viability in cells seeded with these wt and mutant PFFs. In SH-SY5Y cells, all PFFs but G51D induced the cell death (Fig. 4I). NaHS treatment rescued the viability of SH-SY5Y seeded with A53T PFF. H2S slow-releasing agent GYY4137 failed to rescue viability in A53T overexpressing cells seeded with wt PFF (Suppl. Figure 7C). NaHS likely increased cell viability due to suppression of α-Syn oligomerization and increased cellular respiration.

## Discussion

A major obstacle in treatment for PD is the lack of identifiable compounds and therapeutic pathways capable of suppressing alpha-synuclein aggregation. One potential candidate is hydrogen sulfide. It has been shown to play an important role in protecting against organelle (lysosomes, ER, mitochondria) stress^21–23,38^. However, it is not known whether H2S directly affects α-Syn aggregation.

In this study, we examined the effect of H2S donor, NaHS, on alpha-synuclein aggregation. With a pKa of 7.0, at physiological pH, H2S predominantly (72%) exists as a nucleophilic hydrosulfide anion (HS-) species in aqueous solution. The remainder of H2S exists in the form of the dissolved uncharged H2S gas (28%)^39^. However, the solubility of gaseous H2S decreases with increased temperature and increasing concentration of NaCl, resulting in increased uncharged H2S gas in salt solution^40^. H2S is lipophilic and can pass through biological membranes and the hydrophobicity of H2S allows it to partition into the hydrophobic nonpolar core of lipid membranes^41,42^.

α-Syn exists in different conformations^6^. It is inherently unstable in aqueous solution and undergoes aggregation. α-Syn aggregation rate depends on its concentration, pH, temperature, ionic strength, physiochemical properties of amino acid residues, such as hydrophobicity, charge, electrostatic interactions, β sheet-propensity, and mechanical stability of fibrillar structure^43–46^. Alterations in the protein sequence (mutations) affect conformation, contributing to different aggregation propensities. In our study, we performed an examination of fibril formation of wt α-Syn and variants. In α-Syn variants, only A53T and H50Q accelerated the increase in ThT fluorescence in the PFF self-seeded (homotypic) reactions in HEPES buffer (Fig. 1C) whereas the G51D variant was the least efficient. In cross-seeding paradigms, H50Q showed greatest propensity to speed up aggregation of wt and A53T reactions, whereas G51D and A53T were generally inhibitory in cross-seeded paradigms.

Consistent with previous literature, we found that fibrillation kinetics of the A30P mutant seeded with PFF is slower than wildtype at ∼1 mg/ml^47^. The α-Syn mutant A30P has been reported to show different aggregation behavior compared to wt with slightly increased tendency towards β-sheet formation, which could increase its aggregation propensity^48^. It was reported that although A30P α-Syn has a slightly higher initial rate of ThT incorporation, wt and A30P form the same amount of fibrillized material^44^. A30P has also been shown to aggregate slower than wt^49^. The A30P mutation in the N-terminal random coil region, upstream of the hydrophobic Greek-key region, may inhibit interactions with the phospholipid membrane^50^. The A30 residue is located at the center of the N-terminal region and has no amyloidogenicity and low hydropathy. In our study of fibril structure on TEM, A30P formed a branched, interwoven fibril network similar to what was reported previously^49^. Changes were further confirmed by low in vitro aggregation kinetic assembly both in homotypic and heterotypic reactions (Fig. 1B,C), and poor intracellular seeding activity of A30P PFF in α-Syn overexpressing HEK 293 T cells (Fig. 1F–H).

For H50Q, our results are also similar to those found by other groups. α-Syn fibrillization at 5–100 μM concentrations in PBS (pH 7.4) containing 10 μM ThT and incubated at 37 °C and subjected to 900 rpm double orbital shaking demonstrated that the lag phase was shorter for H50Q variant^51^. The kinetics determined by the ThT binding suggested that H50Q α-Syn proteins aggregated through nucleation-dependent polymerization^10^. The lag time for H50Q was shorter than that for wt, indicating that H50Q substantially accelerated the aggregation kinetics in Gly-NaOH buffer (pH 7.4) in the presence of 0.01% sodium azide. In another study, ThT binding kinetics showed that H50Q fibril growth is faster than the wt protein only at concentrations below 45 μM^52^.

A ThT kinetic assay of the G51D fibril formation with and without PFFs (5 mol%) in 50 mM phosphate buffer (pH 7.0) and 50 mM NaCl revealed that in the presence of G51D α-Syn PFF, the G51D α-Syn monomer rapidly forms fibrils, and that wt α-Syn can form the G51D fibril strain in the presence of the preformed G51D fibrils^53^. G51D PFFs also exhibited potent seeding activity by inducing wt α-Syn monomer fibrillization under 50 mM Tris, pH 7.5, 150 mM KCl, 0.05% NaN3 buffer. The lag time for aggregation of 100 μM of most disease mutant monomers into fibrils in 10 mM Tris–HCl buffer (pH 7.4) and 100 mM NaCl (pH 7.4) was comparable to the lag time observed for WT, with only A30P exhibiting a twofold longer lag time^54^. Although the membrane binding affinity and aggregation kinetics of G51D, H50Q, and wt α-Syn monomer were comparable, the ability to permeabilize the membrane differed. Another group showed that in a ThT kinetic assay of wt α-Syn and H50Q and G51Ds mutants in 20 mM phosphate buffer (pH 6.0 and 7.4) containing 0.01% sodium azide at 37 °C with slight agitation, the aggregation kinetics of H50Q were faster compared to the WT, while the rate of amyloid fibril formation was slower in G51D^55^.

Yet another group demonstrated that the elongation rate of α-synuclein at neutral pH was only mildly affected by the disease-associated mutations (A30P, E46K, H50Q, G51D, and A53T)^56^. H50Q, G51D, and A53T variants had the highest elongation rates when the monomers elongated their own seed fibrils. Our results are consistent with the above observations.

The A53 residue is highly amyloidogenic and is located in the hydrophobic pre-NAC region^57,58^. The A53T mutation weakens the hydrophobic packing of the homo-zipper of the preNAC (47GVVHGVTTVA56) thus diminishing its thermodynamic stability^59^. Previous studies support our finding that A53T significantly accelerates fibril formation in comparison to wt, whereas the A30P mutation has a little effect^12^. It should be pointed out that fibril formation in these studies was observed in 20 mM glycine buffer at pH 8.0 and ionic strength and pH significantly influences the kinetics of fibril formation. It was also reported that A53T α-Syn fibrillizes in solution more rapidly than wt in 10 mM Na2HPO4 and 100 mM NaCl at pH 7.4^11^. In HEPES buffer salt buffer salt solution, we observed that A53T α-Syn fibrillizes at the same rate as wt.

Our end-point Quaking-Induced Conversion assay results show that NaHS slowed down A53T α-Syn-A53T α-Syn association in seeded aggregation during the primary nucleation reaction (Fig. 2C,E,F). Intramolecular long-range interactions have been reported between different regions of α-Syn. The β-strands β2-β7 wind around a hydrophobic intra-molecular core composed of alanine and valine residues (i.e. V48, V49, V52, A53, V55, V63, A69, V70, V71, V74, A76, V77, A78, I88, A89, A90, A91)^60^. The hydrophobic core is surrounded by two hydrophilic regions (Q79, T81, and T72, T75, T54, T59, and E61) both still within the core of the structure. The hydrophobic clusters likely contribute to the stability of the protofilament. In A53T α-Syn, introduction of a hydrophilic threonine disrupts the “steric-zipper interphase” between the N- or C-terminal regions and the NAC region that extends and stabilizes β-sheet formation^61^. However, hydrophobic ^40^Val and ^74^Val forms hydrophobic contacts in β-sheet aggregation in the hydrophobic regions 36–41 and 68–78, leading to A53T α-Syn fibrilization^62^. The hydrophobic effect occurs via displacement of water molecules from around the hydrophobic surfaces. H2S permeates membranes like a hydrophobic solute since it is a slightly polar molecule^53,54,63,64^.

The ability of H2S to permeate hydrophobic regions and its slightly polar nature may explain the inhibitory effects on α-Syn aggregation. NaHS, exhibited little effect on wt fibrilization but significantly inhibited A53T mutant fibrilization. These results suggest that HS-/H2S may reach hydrophobic regions and prevent hydrophobic contact of ^40^Val and ^74^Val but not alanine and valine residues. In a polar environment (e.g. a buffer), hydrophobic patches interact with hydrophobic patches of other molecules to form aggregates. However, hydrophobic and less polar solutes contribute to changes in bulk properties of the solvent and solvent-mediated interactions leading to suppression of fibril formation^65^. For aggregation of A53T monomer with wt PFF, we observed a shift in initial lag phase in the presence of NaHS followed by a continuous structural development of fibrils until reaching an apparent monomer-aggregate equilibrium state and a plateau at lower Thioflavin T (ThT) fluorescence intensity (Fig. 2C). In the presence of NaHS, the shift in initial lag phase was associated with significant fibril truncation (Fig. 2B,E).

Previous studies have found that oligomers promote the production of reactive oxygen species (ROS) and that oligomers may induce ROS in neuronal culture^66,67^. Two characteristics of H2S-reactive oxygen species quenching properties and stimulation of cellular enzymatic or nonenzymatic antioxidants to scavenge free radicals explain at least part of the observed protective behavior of NaHS^18,24,68^. Aggregated α-Syn accumulates intracellularly in cytoplasm and cell organelles and causes cellular dysfunction and death^69,70^. PD familial mutation A53T but not A30P enhances the propagation, mitochondrial fragmentation and cytotoxicity of α-Syn fibrils^71,72^. In vitro experiments show that A30P and G51D delay intracellular α-Syn aggregation^73^.

Although it was previously shown that H50Q fibrils had higher seeding capacity in HEK 293 T A53T-YFP biosensor cells, we did not observe similar seeding efficiency of H50Q^74^. This may be due to differences in the concentration of PFFs used for the seeding. Boyer et al. used a range of concentrations from 20–200 nM fibrils. However, the authors observed lower MTT toxicity of H50Q in PC12 cells. H2S has cytoprotective activity, which can protect against α-Syn aggregation-induced cell death. H2S stimulates mitochondrial electron transport, prevents mitochondria swelling and decreases oxidative stress^21^.

The observed protective effect of H2S donor NaHS has traditionally been attributed to anti-oxidant and anti-inflammatory effects. Our data identify another potential cytoprotective mechanism of H2S as direct inhibition of intracellular α-Syn aggregation. Analysis of intracellular α-Syn oligomers showed that treatment with NaHS decreased aggregation propensity of α-Syn, and specifically reduced intracellular accumulation and cytotoxicity. This mechanism was conformation dependent. Further investigations into H2S releasing agents and their mechanism of action may yield useful future therapeutics for PD.

## Materials and methods

### Materials

Sodium hydrosulfide, DTT, ampicillin, EDTA, sucrose, Tris–HCl, proteinase K, from Sigma Aldrich, restriction enzymes from New England Biolabs (NEB), poly-D-lysine, Opti-MEM, Bacto Yeast and Bacto Trypton from Gibco, SOC and Lipofectamine 2000 from Invitrogen by Thermo Fisher Scientific, Criterion TGX gels, sample buffer and precision plus protein standards from Bio-Rad, QuickChange II site-directed mutagenesis kit and E. coli BL21 (DE3) from Agilent, Recombinant Anti-Alpha-synuclein antibody MJFR1 from Abcam (cat. Ab138501, Lot 1,001,572–18), Purified Mouse Anti-α-Synuclein Clone 42/α-Synuclein from BD Biosciences (cat. 610,787, Lot 3,268,708) paraformaldehyde 16% w/v and formvar/carbon film 300 mesh from Electron microscopy sciences.

## Methods

### Site-directed mutagenesis

PRK 172 wt α-Syn gene fragments served as mutagenesis templates. Site-directed mutations were introduced at specific positions (A30P, H50Q, G51D, and A53T) using QuikChange site-directed mutagenesis kit (Agilent) and using designed primers α-Syn-A30P-F: ggt gtg gca gaa gca cca gga aag aca aaa gag and α-Syn-A30P-R : ctc ttt tgt ctt tcc tgg tgc ttc tgc cac acc, α-Syn-H50Q-F : aag gag gga gtg gtg caa ggt gtg gca aca gtg and α-Syn-H50Q-R : cac tgt tgc cac acc ttg cac cac tcc ctc ctt, α-Syn-G51D-F: gag gga gtg gtg cat gat gtg gca aca gtg gct and α-Syn-G51D-R: agc cac tgt tgc cac atc atg cac cac tcc ctc, α-Syn-A53T-F: gtg gtg cat ggt gtg aca aca gtg gct gag aag and α-Syn-A53T-R: ctt ctc agc cac tgt tgt cac acc atg cac cac. In total, 50 µl PCR reaction was carried out with 50 ng DNA templates, 125 ng primer pair, 200 mM dNTPs, and 2 U of pfu DNA polymerase. The PCR amplification products were evaluated by 1% agarose gel electrophoresis in TAE buffer. The PCRs were incubated with restriction enzyme DpnI (NEB) at 37°C for 1 h to digest the parental supercoiled dsDNA. An aliquot of 1 µl Dpn I-treated PCR product was transformed into XL-1 blue competent E. coli cells and inoculated on Luria–Bertani (LB) plate containing 50 mg/mL ampicillin. All mutations were confirmed by DNA sequencing (Eurofins MWG Operon). Alpha-synuclein WT and mutant sequencing results for this project are available in GenBank National Libraries of Medicine (NLM) under the following Accession numbers and links:

- PV034807 (A30P) https://www.ncbi.nlm.nih.gov/search/all/?term=PV034807
- PV034808 (H50Q) https://www.ncbi.nlm.nih.gov/search/all/?term=PV034808
- PV034809 (A53T) https://www.ncbi.nlm.nih.gov/search/all/?term=PV0348
- PV034810 (WT) https://www.ncbi.nlm.nih.gov/search/all/?term=PV034810
- PV034811(G51D) https://www.ncbi.nlm.nih.gov/search/all/?term=PV034811

### Purification of recombinant alpha-synuclein

Human α-syn proteins, either wild-type or with mutation at alanine 30 (A30P), histidine 50 (H50Q), glycine 51 (G51D), and alanine 53 (A53T), were purified from Escherichia coli (E. coli) as described previously^75^. Briefly, a plasmid containing WT human αSyn was expressed in E. coli BL21 (DE3) (Agilent). The cells were suspended in osmotic shock buffer (30 mM Tris–HCl, 2 mM EDTA, 40% sucrose, pH 7.2), centrifuged. Streptomycin sulfate (final 10 mg/ml) was added to the supernatant and centrifuged. After addition of DTT (final 1 mM) and Tris–HCl (final 20 mM, pH 8.0) solutions supernatant was then heated at 100 °C in a water bath and centrifuged. The supernatant was applied onto a DEAE Sepharose beads Poly-Prep chromatography column equilibrated with 20 mM Tris–HCl (pH 8.0), 1 mM EDTA and 1 mM DTT. The purified α-Syn was dialyzed against Tris–HCl/NaCl/DTT at 4 °C overnight and stored at − 80 °C. Protein concentration of α-Syn monomer was determined using Micro BCA protein assay (ThermoScientific).

### In vitro aggregation assay

The in vitro fibrillation assay was performed as reported previously^75^. Purified recombinant stocks of α- Syn monomer in 20 mM Tris–HCl/100 mM NaCl buffer pH 8.0 were prepared to a final concentration of 2 mg/ml. Samples were incubated for 3 days in a Thermomixer (Eppendorf) with shaking at 1000 rpm and 37 °C. To determine the concentration of fibrils, the reaction mix was centrifuges for 15 min at 15,000 g to separate fibrils from monomer. Protein concentration of α-Syn monomer was determined using Micro BCA protein assay (ThermoScientific). Pelleted fibrils were resuspended in 20 mM Tris–HCl buffer and stored at − 80 °C until further use. further use.

### Endpoint quaking-induced conversion (EP-QuIC) Thioflavin-T-based kinetic aggregation assays

EP-QuIC was performed as described previously^31,32^ with modifications. Briefly, seeded α-Syn aggregation was conducted in 10 mM HEPES (pH 7.4) with 400 mM NaCl, 0.0125% SDS containing 1 mg/ml recombinant full-length human α-syn protein and 10 μM thioflavin T (ThT) in 96-well black plates with clear bottom (Nalgene Nunc). Recombinant human α-syn proteins, either wild-type or with mutation at alanine 30 (A30P), histidine 50 (H50Q), glycine 51 (G51D), and alanine 53 (A53T) were utilized. The pre- formed fibrils (PFF) were stored at − 80 °C. Prior to use, PFFs were thawed on ice and then sonicated for 3 min at 65A. Subsequently, sonicated PFF were used as seeds at 1 and 10 nM concentration. Reactions for individual samples were prepared in triplicate. The plates were placed in an Eppendorf Thermomixer C equipped with a Smart Block plate and with a heated ThermoTop with shaking cycles consisting of 1 min of circular shaking at 700 rpm and 1 min of rest at 45 °C. After 60 min, the plates were removed from the Eppendorf Thermomixer C, and an RFU reading was taken on a BioTek Citation 3 Hybrid Multi-Mode plate reader using 440 ± 15 nm excitation and 480 ± 15 nm emission (bottom read). The plate was returned to the Eppendorf Thermomixer C, shaking cycles continued for another 60 min with next reading taken on a BioTek Citation 3 plate reader. Plates were measured every 60 min for 8 to 10 h with circular shaking. In the present study, the time-to-threshold (lag time) was determined for each reaction as the earliest time point after the fluorescence signal exceeded ThT threshold value. NaHS was added to the reaction at concentration of 1 µM prior adding PFF.

### AmyloFit 2.0 analysis

We used the AmyloFit tool at (https://amylofit.com/amylofitmain/fitter/) which has been utilized in published literature^37^ to fit the results of QuIC reactions into the secondary nucleation model to calculate the K_+_, which denotes the aggregation rate constant for growth in the absence and presence of inhibitor with a mean residual error of less than 0.002.

### Transmission electron microscopy (TEM)

For TEM imaging, wt α-Syn and PD pathogenic missense variants samples were diluted in Milli-Q water to a final concentration of 1 μM. Copper grids were glow discharged for 30–60 s. 10 µl of samples were absorbed onto carbon-coated copper grids, washed with water and negatively stained with 1% (w/v) uranyl acetate for 3 min. The grids were dried for at least 20 min before analysis. Micrographs were collected with a Thermo Scientific™ Talos™ F200X TEM operating at 200 kV accelerating voltage using a 16 M pixel 4 k x 4 k CMOS camera (Thermo Scientific™ Ceta). 50 µm objective aperture was inserted while imaging to enhance contrast. TEM image defocus was between − 1 µm under-focus, in-focus, + 1 µm over-focus.

### Proteinase K digestion

Real time quaking-induced conversion (EP-QuIC) α-Syn samples were incubated with 4 µg/ml proteinase K (PK) (Sigma Aldrich) at 37 °C. Proteolytic aliquots were taken at different time points (0, 5, 15, and 30 min) and the reactions were quenched by addition of 4 times concentrated denaturing loading buffer (8 µl of 4 × loading buffer to 25 µl reaction). There were at least 3 technical replicates for each experimental condition. Samples were heated at 100 °C for 10 min, and 10 μL of each sample was loaded on Criterion SDS-PAGE gels and stained with QC Colloidal Coomassie Stain.

### Native dot blot analysis

Bio-Dot microfiltration apparatus was used. QuIC products were spotted on nitrocellulose membrane. Membranes were blocked in 5% skimmed milk in TBST for 1 h at room temperature and then were incubated overnight at 4 °C with primary monoclonal αSyn antibody (BD Bioscience) in a dilution of 1:1,000. On the following day, membranes were washed in TBST and incubated with HRP-conjugated secondary antibody for 1 h at room temperature. Membranes were developed with SuperSignal West Femto Maximum Sensitivity Substrate (Thermo Scientific) and processed using ChemiDoc MP Imaging System (Bio-Rad). The optical density was analyzed using Image Lab software.

The antibodies, purified Mouse Anti-α-Synuclein Clone 42/α-Synuclein antibody from BD Biosciences (cat. 610,787, Lot 3,268,708) and MJFR1 from Abcam (cat. Ab138501, Lot 1,001,572–18), were validated in numerous studies including Diamond M.I.^35^ and Prots I.^76^ groups.

### Cells plating and seeding with PFF

HEK 293 T, HEK 293 T A53T-YFP, HEK 293 T wt-YFP, and SH-SY5Y cells were plated on poly-lysine treated glass slides and culture in Dulbecco’s Modified Eagle Medium (DMEM) supplemented with 10% fetal bovine serum (FBS) and 1% penicillin and streptomycin (P/S) at 37 °C and atmosphere with 5% CO2. After 24 h, cells were treated with NaHS and then cells were seeded with recombinant preformed fibrils. Fibrils were sonicated for 3 min at 65A (Qsonica 700) on ice. Samples for seeding were prepared in Opti-MEM (Gibco) with 1 μl of Lipofectamine 2000 (Invitrogen) to a total volume of 20 μl/well and incubated at room temperature for 30 min. Samples were added dropwise to wells. The incubation was for 24, 48 and 72 h prior to imaging.

HEK 293 T cells overexpressing A53T α-Syn labelled with YFP (HEK 293 T A53T-YFP) and HEK 293 T cells labelled with A53T α-Syn labelled with CFP and YFP (HEK 293 T-CFP/YFP) were obtained from the lab of Diamond M.I. at Washington University St Louis where they were first generated and characterized^35^. We used anti-α-Syn MJFR1 Ab from Abcam (cat. Ab138501, Lot 1,001,572–18) and anti-α-Syn [MJFR-14–6-4–2] (abcam cat. Ab209538).

### Super-resolution imaging and analysis

Following the seeding HEK 293 T A53T-YFP cells were washed with PBS and fixed at room temperature for 20 min with a final concentration of 2% paraformaldehyde (PFA). Then cells were washed with PBS and imaged using N-SIM S Super-Resolution Microscope (Nikon). The images were analyzed with the Imaris software (BitPlane, Oxford Instruments).

### Immunofluorescence, FRET and image analysis

HEK 293 T A53T-CFP/YFP cells were treated with NaHS and seeded with 1 and 10 nM preformed fibrils. After 24 h, cells were fixed at room temperature for 20 min with a final concentration of 2% paraformaldehyde (PFA). Then cells were washed with PBS and imaged under a confocal microscope Nikon AX-R (Nikon) at an excitation at 405 and 488. FRET efficiency was assessed using a Fret Analyzer plugin for ImageJ program (https://imagej.net/ij/plugins/fret-analyzer/fret-analyzer.htm). For FRET analysis, a stack of three images of the group of cells at excitation of the YFP and detection of CFP, excitation of YFP and detection of YFP, and excitation of CFP and detection of CFP were analyzed. For donor bleed-through, one stack of two images of HEK 293 T A53T-CFP cells was analyzed and for acceptor bleed-through images of HEK 293 T A53T- YFP cells were analyzed.

### XTT viability assay

XTT assay was performed following method described by Roehm et al.^77^. In brief, XTT (3’-[1-[(phenylamino)-carbonyl]-3,4-tetrazolium]-bis(4-metoxy-6-nitro)benzene-sulfonic acid sodium salt) was made fresh each day by dissolving XTT in PBS at 1 mg/ml, PMS (phenazine methosulfate, Sigma P9625-1 g, Mr = 306.3) was made up as a 100 mM stock solution in H2O. XTT/PMS solution was prepared before use by mixing 100 ul of 1 mg/ml XTT and 2.5 ul of 5 mM PMS. 25 µl of XTT/PMS solution was added to each 100 µl culture medium giving a final concentration of 0.2 mg/ml XTT and 25 µM PMS. After 2 h of incubation at 37 °C the absorbance was measured using a microplate reader at a test wavelength of 450 nm and reference wavelength of 650 nm. The % damage was calculated utilizing (1-A_test_/A_control_) × 100. All data were expressed as mean ± standard deviation. Comparisons between the different groups were performed by one-way ANOVA using GrapPad Prism.

### Statistical analysis

The data shown for each experiment were based on at least three technical replicates, as indicated in the individual figure legends. The data are presented as the means ± SDs, and *p* values were determined using using one-way ANOVA statistics, followed by Dunnett’s multiple comparison test built into GraphPad Prism 9.5.0 were appropriate. The differences were considered significant when *p < 0.05, **p < 0.005, ***p < 0.0005. ****p < 0.0001.

## Electronic supplementary material

Below is the link to the electronic supplementary material.

Supplementary Material 1

## Data Availability

The DNA sequences used and/or analyzed during the current study were uploaded to the National Library of Medicine GenBank. Sequencing results for alpha-synuclein WT and mutant utilized in this project are available under the following accession numbers and links: BankIt2919895 PV034807 (A30P) https://www.ncbi.nlm.nih.gov/search/all/?term=PV034807. BankIt2919900 PV034808 (H50Q) https://www.ncbi.nlm.nih.gov/search/all/?term=PV034808. BankIt2919902 PV034809 (A53T) https://www.ncbi.nlm.nih.gov/search/all/?term=PV034809. BankIt2919903 PV034810 (WT) https://www.ncbi.nlm.nih.gov/search/all/?term=PV034810. BankIt2919904 PV034811(G51D) https://www.ncbi.nlm.nih.gov/search/all/?term=PV034811

## References

[1] Samii, A., Nutt, J. G. & Ransom, B. R. Parkinson’s disease. *Lancet* **363**, 1783–1793. 10.1016/S0140-6736(04)16305-8 (2004). 15172778 10.1016/S0140-6736(04)16305-8

[2] Bloem, B. R., Okun, M. S. & Klein, C. Parkinson’s disease. *Lancet* **397**, 2284–2303. 10.1016/S0140-6736(21)00218-X (2021). 33848468 10.1016/S0140-6736(21)00218-X

[3] Collaborators, G. B. D. Global, regional, and national burden of Parkinson’s disease, 1990–2016: A systematic analysis for the Global Burden of Disease Study 2016. *Lancet Neurol* **17**, 939–953 (2018). 10.1016/S1474-4422(18)30295-3 10.1016/S1474-4422(18)30295-3 PMC6191528 30287051

[4] Ou, Z. et al. Global trends in the incidence, prevalence, and years lived with disability of Parkinson’s disease in 204 countries/territories from 1990 to 2019. *Front. Public Health* **9**, 776847. 10.3389/fpubh.2021.776847 (2021). 34950630 10.3389/fpubh.2021.776847 PMC8688697

[5] Willis, A. W. et al. Incidence of Parkinson disease in North America. *NPJ Parkinsons Dis.* **8**, 170. 10.1038/s41531-022-00410-y (2022). 36522332 10.1038/s41531-022-00410-y PMC9755252

[6] Fauvet, B. et al. alpha-Synuclein in central nervous system and from erythrocytes, mammalian cells, and Escherichia coli exists predominantly as disordered monomer. *J. Biol. Chem.* **287**, 15345–15364. 10.1074/jbc.M111.318949 (2012). 22315227 10.1074/jbc.M111.318949 PMC3346117

[7] Smaldone, G. et al. Insight into conformational modification of alpha-synuclein in the presence of neuronal whole cells and of their isolated membranes. *FEBS Lett.* **589**, 798–804. 10.1016/j.febslet.2015.02.012 (2015). 25701590 10.1016/j.febslet.2015.02.012

[8] Mahul-Mellier, A. L. et al. The process of Lewy body formation, rather than simply alpha-synuclein fibrillization, is one of the major drivers of neurodegeneration. *Proc. Natl. Acad. Sci. USA* **117**, 4971–4982. 10.1073/pnas.1913904117 (2020). 32075919 10.1073/pnas.1913904117 PMC7060668

[9] Fares, M. B. et al. The novel Parkinson’s disease linked mutation G51D attenuates in vitro aggregation and membrane binding of alpha-synuclein, and enhances its secretion and nuclear localization in cells. *Hum. Mol. Genet.* **23**, 4491–4509. 10.1093/hmg/ddu165 (2014). 24728187 10.1093/hmg/ddu165 PMC4119404

[10] Ghosh, D. et al. The Parkinson’s disease-associated H50Q mutation accelerates alpha-Synuclein aggregation in vitro. *Biochemistry* **52**, 6925–6927. 10.1021/bi400999d (2013). 24047453 10.1021/bi400999d

[11] Conway, K. A., Harper, J. D. & Lansbury, P. T. Accelerated in vitro fibril formation by a mutant alpha-synuclein linked to early-onset Parkinson disease. *Nat. Med.* **4**, 1318–1320. 10.1038/3311 (1998). 9809558 10.1038/3311

[12] Ohgita, T., Namba, N., Kono, H., Shimanouchi, T. & Saito, H. Mechanisms of enhanced aggregation and fibril formation of Parkinson’s disease-related variants of alpha-synuclein. *Sci. Rep.* **12**, 6770. 10.1038/s41598-022-10789-6 (2022). 35474118 10.1038/s41598-022-10789-6 PMC9043213

[13] Das, D. & Mattaparthi, V. S. K. Conformational dynamics of A30G alpha-synuclein that causes familial Parkinson disease. *J. Biomol. Struct. Dyn* **41**, 14702–14714. 10.1080/07391102.2023.2193997 (2023). 36961209 10.1080/07391102.2023.2193997

[14] Ono, K., Tsuji, M., Yamasaki, T. R. & Pasinetti, G. M. Anti-aggregation effects of phenolic compounds on alpha-synuclein. *Molecules*10.3390/molecules25102444 (2020). 32456274 10.3390/molecules25102444 PMC7288075

[15] Singh, P. K. et al. Curcumin modulates alpha-synuclein aggregation and toxicity. *ACS Chem. Neurosci.* **4**, 393–407. 10.1021/cn3001203 (2013). 23509976 10.1021/cn3001203 PMC3605819

[16] Wang, Q., Guo, J., Jiao, P., Liu, H. & Yao, X. Exploring the influence of EGCG on the beta-sheet-rich oligomers of human islet amyloid polypeptide (hIAPP1-37) and identifying its possible binding sites from molecular dynamics simulation. *PLoS ONE* **9**, e94796. 10.1371/journal.pone.0094796 (2014). 24739876 10.1371/journal.pone.0094796 PMC3989243

[17] Wang, Y. et al. Epigallocatechin-3-gallate: A phytochemical as a promising drug candidate for the treatment of Parkinson’s disease. *Front. Pharmacol.* **13**, 977521. 10.3389/fphar.2022.977521 (2022). 36172194 10.3389/fphar.2022.977521 PMC9511047

[18] Kimura, H. Hydrogen sulfide and polysulfides as signaling molecules. *Proc. Jpn. Acad. Ser. B Phys. Biol. Sci.* **91**, 131–159. 10.2183/pjab.91.131 (2015). 25864468 10.2183/pjab.91.131 PMC4568289

[19] Cirino, G., Szabo, C. & Papapetropoulos, A. Physiological roles of hydrogen sulfide in mammalian cells, tissues, and organs. *Physiol. Rev.* **103**, 31–276. 10.1152/physrev.00028.2021 (2023). 35435014 10.1152/physrev.00028.2021

[20] Pagani, S. & Galante, Y. M. Interaction of rhodanese with mitochondrial NADH dehydrogenase. *Biochim. Biophys. Acta* **742**, 278–284. 10.1016/0167-4838(83)90312-6 (1983). 6402020 10.1016/0167-4838(83)90312-6

[21] Paul, B. D., Snyder, S. H. & Kashfi, K. Effects of hydrogen sulfide on mitochondrial function and cellular bioenergetics. *Redox Biol.* **38**, 101772. 10.1016/j.redox.2020.101772 (2021). 33137711 10.1016/j.redox.2020.101772 PMC7606857

[22] Wang, H., Shi, X., Qiu, M., Lv, S. & Liu, H. Hydrogen sulfide plays an important protective role through influencing endoplasmic reticulum stress in diseases. *Int. J. Biol. Sci.* **16**, 264–271. 10.7150/ijbs.38143 (2020). 31929754 10.7150/ijbs.38143 PMC6949148

[23] Zhang, Y. et al. Golgi stress response, hydrogen sulfide metabolism, and intracellular calcium homeostasis. *Antioxid. Redox Signal* **32**, 583–601. 10.1089/ars.2019.7824 (2020). 31870162 10.1089/ars.2019.7824

[24] Murphy, B., Bhattacharya, R. & Mukherjee, P. Hydrogen sulfide signaling in mitochondria and disease. *FASEB J.* **33**, 13098–13125. 10.1096/fj.201901304R (2019). 31648556 10.1096/fj.201901304R PMC6894098

[25] Tabassum, R., Jeong, N. Y. & Jung, J. Protective effect of hydrogen sulfide on oxidative stress-induced neurodegenerative diseases. *Neural Regen. Res.* **15**, 232–241. 10.4103/1673-5374.265543 (2020). 31552888 10.4103/1673-5374.265543 PMC6905340

[26] Shefa, U., Kim, M. S., Jeong, N. Y. & Jung, J. Antioxidant and cell-signaling functions of hydrogen sulfide in the central nervous system. *Oxid. Med. Cell Longev.* **2018**, 1873962. 10.1155/2018/1873962 (2018). 29507650 10.1155/2018/1873962 PMC5817206

[27] Zhao, F. L. et al. Hydrogen sulfide selectively inhibits gamma-secretase activity and decreases mitochondrial abeta production in neurons from APP/PS1 transgenic mice. *Neurochem. Res.* **41**, 1145–1159. 10.1007/s11064-015-1807-7 (2016). 26708452 10.1007/s11064-015-1807-7

[28] Hou, X. et al. GYY4137, an H(2)S slow-releasing donor, prevents nitrative stress and alpha-synuclein nitration in an MPTP mouse model of Parkinson’s disease. *Front. Pharmacol.* **8**, 741. 10.3389/fphar.2017.00741 (2017). 29163149 10.3389/fphar.2017.00741 PMC5671206

[29] Liu, L., Wang, J. & Wang, H. Hydrogen sulfide alleviates oxidative stress injury and reduces apoptosis induced by MPP(+) in Parkinson’s disease cell model. *Mol. Cell Biochem.* **472**, 231–240. 10.1007/s11010-020-03801-y (2020). 32577946 10.1007/s11010-020-03801-y

[30] Lee, Z. W. et al. The slow-releasing hydrogen sulfide donor, GYY4137, exhibits novel anti-cancer effects in vitro and in vivo. *PLoS ONE* **6**, e21077. 10.1371/journal.pone.0021077 (2011). 21701688 10.1371/journal.pone.0021077 PMC3119065

[31] Cheng, K. et al. Endpoint quaking-induced conversion: a sensitive, specific, and high-throughput method for antemortem diagnosis of Creutzfeldt–Jacob disease. *J. Clin. Microbiol.* **54**, 1751–1754. 10.1128/JCM.00542-16 (2016). 27076662 10.1128/JCM.00542-16 PMC4922112

[32] Kaelber, N., Bett, C., Asher, D. M. & Gregori, L. Quaking-induced conversion of prion protein on a thermal mixer accelerates detection in brains infected with transmissible spongiform encephalopathy agents. *PLoS ONE* **14**, e0225904. 10.1371/journal.pone.0225904 (2019). 31830760 10.1371/journal.pone.0225904 PMC6908438

[33] Jarrett, J. T. & Lansbury, P. T. Jr. Seeding “one-dimensional crystallization” of amyloid: A pathogenic mechanism in Alzheimer’s disease and scrapie?. *Cell* **73**, 1055–1058. 10.1016/0092-8674(93)90635-4 (1993). 8513491 10.1016/0092-8674(93)90635-4

[34] Xu, B. et al. Distinct effects of familial parkinson’s disease-associated mutations on alpha-synuclein phase separation and amyloid aggregation. *Biomolecules*10.3390/biom13050726 (2023). 37238596 10.3390/biom13050726 PMC10216457

[35] Yamasaki, T. R. et al. Parkinson’s disease and multiple system atrophy have distinct alpha-synuclein seed characteristics. *J. Biol. Chem.* **294**, 1045–1058. 10.1074/jbc.RA118.004471 (2019). 30478174 10.1074/jbc.RA118.004471 PMC6341389

[36] Han, Y., Shang, Q., Yao, J. & Ji, Y. Hydrogen sulfide: a gaseous signaling molecule modulates tissue homeostasis: implications in ophthalmic diseases. *Cell Death Dis.* **10**, 293. 10.1038/s41419-019-1525-1 (2019). 30926772 10.1038/s41419-019-1525-1 PMC6441042

[37] Meisl, G. et al. Molecular mechanisms of protein aggregation from global fitting of kinetic models. *Nat. Protoc.* **11**, 252–272. 10.1038/nprot.2016.010 (2016). 26741409 10.1038/nprot.2016.010

[38] Gilbert, A. K. & Pluth, M. D. Subcellular delivery of hydrogen sulfide using small molecule donors impacts organelle stress. *J. Am. Chem. Soc.* **144**, 17651–17660. 10.1021/jacs.2c07225 (2022). 36121306 10.1021/jacs.2c07225 PMC9896967

[39] Li, Q. & Lancaster, J. R. Jr. Chemical foundations of hydrogen sulfide biology. *Nitric Oxide* **35**, 21–34. 10.1016/j.niox.2013.07.001 (2013). 23850631 10.1016/j.niox.2013.07.001 PMC3843984

[40] Barrett, T. J., Anderson, G. M. & Lugowski, J. The solubility of hydrogen-sulfide in 0–5 M Nacl solutions at 25-degrees-95-degrees-C and one atmosphere. *Geochim. Cosmochim. Acta* **52**, 807–811 (1988). 10.1016/0016-7037(88)90352-3

[41] Cuevasanta, E., Denicola, A., Alvarez, B. & Moller, M. N. Solubility and permeation of hydrogen sulfide in lipid membranes. *PLoS ONE* **7**, e34562. 10.1371/journal.pone.0034562 (2012). 22509322 10.1371/journal.pone.0034562 PMC3324494

[42] Riahi, S. & Rowley, C. N. Why can hydrogen sulfide permeate cell membranes?. *J Am Chem Soc* **136**, 15111–15113. 10.1021/ja508063s (2014). 25323018 10.1021/ja508063s

[43] Chiti, F., Stefani, M., Taddei, N., Ramponi, G. & Dobson, C. M. Rationalization of the effects of mutations on peptide and protein aggregation rates. *Nature* **424**, 805–808. 10.1038/nature01891 (2003). 12917692 10.1038/nature01891

[44] Chiti, F. et al. Kinetic partitioning of protein folding and aggregation. *Nat. Struct. Biol.* **9**, 137–143. 10.1038/nsb752 (2002). 11799398 10.1038/nsb752

[45] Lins, L. & Brasseur, R. The hydrophobic effect in protein folding. *FASEB J.* **9**, 535–540. 10.1096/fasebj.9.7.7737462 (1995). 7737462 10.1096/fasebj.9.7.7737462

[46] Wiggins, P. M. Hydrophobic hydration, hydrophobic forces and protein folding. *Physica A* **238**, 113–128 (1997). 10.1016/S0378-4371(96)00431-1

[47] Li, J., Uversky, V. N. & Fink, A. L. Conformational behavior of human alpha-synuclein is modulated by familial Parkinson’s disease point mutations A30P and A53T. *Neurotoxicology* **23**, 553–567. 10.1016/s0161-813x(02)00066-9 (2002). 12428728 10.1016/s0161-813x(02)00066-9

[48] Li, J., Uversky, V. N. & Fink, A. L. Effect of familial Parkinson’s disease point mutations A30P and A53T on the structural properties, aggregation, and fibrillation of human alpha-synuclein. *Biochemistry* **40**, 11604–11613. 10.1021/bi010616g (2001). 11560511 10.1021/bi010616g

[49] Nielsen, S. B. et al. Wildtype and A30P mutant alpha-synuclein form different fibril structures. *PLoS ONE* **8**, e67713. 10.1371/journal.pone.0067713 (2013). 23861789 10.1371/journal.pone.0067713 PMC3701545

[50] Kruger, R. et al. Ala30Pro mutation in the gene encoding alpha-synuclein in Parkinson’s disease. *Nat. Genet.* **18**, 106–108. 10.1038/ng0298-106 (1998). 9462735 10.1038/ng0298-106

[51] Porcari, R. et al. The H50Q mutation induces a 10-fold decrease in the solubility of alpha-synuclein. *J. Biol. Chem.* **290**, 2395–2404. 10.1074/jbc.M114.610527 (2015). 25505181 10.1074/jbc.M114.610527 PMC4303689

[52] Khalaf, O. et al. The H50Q mutation enhances alpha-synuclein aggregation, secretion, and toxicity. *J. Biol. Chem.* **289**, 21856–21876. 10.1074/jbc.M114.553297 (2014). 24936070 10.1074/jbc.M114.553297 PMC4139205

[53] Sun, Y. et al. The hereditary mutation G51D unlocks a distinct fibril strain transmissible to wild-type alpha-synuclein. *Nat. Commun.* **12**, 6252. 10.1038/s41467-021-26433-2 (2021). 34716315 10.1038/s41467-021-26433-2 PMC8556266

[54] Stefanovic, A. N., Lindhoud, S., Semerdzhiev, S. A., Claessens, M. M. & Subramaniam, V. Oligomers of Parkinson’s disease-related alpha-synuclein mutants have similar structures but distinctive membrane permeabilization properties. *Biochemistry* **54**, 3142–3150. 10.1021/bi501369k (2015). 25909158 10.1021/bi501369k

[55] Ranjan, P. & Kumar, A. Perturbation in long-range contacts modulates the kinetics of amyloid formation in alpha-synuclein familial mutants. *ACS Chem. Neurosci.* **8**, 2235–2246. 10.1021/acschemneuro.7b00149 (2017). 28759722 10.1021/acschemneuro.7b00149

[56] Flagmeier, P. et al. Mutations associated with familial Parkinson’s disease alter the initiation and amplification steps of alpha-synuclein aggregation. *Proc. Natl. Acad. Sci. USA* **113**, 10328–10333. 10.1073/pnas.1604645113 (2016). 27573854 10.1073/pnas.1604645113 PMC5027465

[57] Rodriguez, J. A. et al. Structure of the toxic core of alpha-synuclein from invisible crystals. *Nature* **525**, 486–490. 10.1038/nature15368 (2015). 26352473 10.1038/nature15368 PMC4791177

[58] Doherty, C. P. A. et al. A short motif in the N-terminal region of alpha-synuclein is critical for both aggregation and function. *Nat. Struct. Mol. Biol.* **27**, 249–259. 10.1038/s41594-020-0384-x (2020). 32157247 10.1038/s41594-020-0384-x PMC7100612

[59] Li, B. et al. Cryo-EM of full-length alpha-synuclein reveals fibril polymorphs with a common structural kernel. *Nat. Commun.* **9**, 3609. 10.1038/s41467-018-05971-2 (2018). 30190461 10.1038/s41467-018-05971-2 PMC6127345

[60] Guerrero-Ferreira, R. et al. Cryo-EM structure of alpha-synuclein fibrils. *Elife*10.7554/eLife.36402 (2018). 29969391 10.7554/eLife.36402 PMC6092118

[61] Coskuner, O. & Wise-Scira, O. Structures and free energy landscapes of the A53T mutant-type alpha-synuclein protein and impact of A53T mutation on the structures of the wild-type alpha-synuclein protein with dynamics. *ACS Chem. Neurosci.* **4**, 1101–1113. 10.1021/cn400041j (2013). 23607785 10.1021/cn400041j PMC3715894

[62] Kumar, S., Sarkar, A. & Sundar, D. Controlling aggregation propensity in A53T mutant of alpha-synuclein causing Parkinson’s disease. *Biochem. Biophys. Res. Commun.* **387**, 305–309. 10.1016/j.bbrc.2009.07.008 (2009). 19580781 10.1016/j.bbrc.2009.07.008

[63] Krupp, R. E. in *Goldschmidt Conference* (Edinburgh, 1994).

[64] Sun, L. & Liang, J. R. Solubility modeling of hydrogen sulfide in aqueous sodium salt solutions. *Environ. Technol. Innov.*10.1016/j.eti.2023.103334 (2023).

[65] Bucciarelli, S. et al. Disentangling the role of solvent polarity and protein solvation in folding and self-assembly of alpha-lactalbumin. *J. Colloid Interface Sci.* **561**, 749–761. 10.1016/j.jcis.2019.11.051 (2020). 31771874 10.1016/j.jcis.2019.11.051

[66] Cremades, N. et al. Direct observation of the interconversion of normal and toxic forms of alpha-synuclein. *Cell* **149**, 1048–1059. 10.1016/j.cell.2012.03.037 (2012). 22632969 10.1016/j.cell.2012.03.037 PMC3383996

[67] Iljina, M. et al. Kinetic model of the aggregation of alpha-synuclein provides insights into prion-like spreading. *Proc. Natl. Acad. Sci. USA* **113**, E1206-1215. 10.1073/pnas.1524128113 (2016). 26884195 10.1073/pnas.1524128113 PMC4780632

[68] Lin, K. et al. Hydrogen sulfide can scavenge free radicals to improve spinal cord injury by inhibiting the p38MAPK/mTOR/NF-kappaB signaling pathway. *Neuromol. Med.* **26**, 26. 10.1007/s12017-024-08794-1 (2024). 10.1007/s12017-024-08794-1 38907170

[69] Prymaczok, N. C. et al. Cell-to-cell transmitted alpha-synuclein recapitulates experimental Parkinson’s disease. *NPJ Parkinsons Dis.* **10**, 10. 10.1038/s41531-023-00618-6 (2024). 38184623 10.1038/s41531-023-00618-6 PMC10771530

[70] Wang, X. et al. Pathogenic alpha-synuclein aggregates preferentially bind to mitochondria and affect cellular respiration. *Acta Neuropathol. Commun.* **7**, 41. 10.1186/s40478-019-0696-4 (2019). 30871620 10.1186/s40478-019-0696-4 PMC6419482

[71] Nakamura, K. et al. Direct membrane association drives mitochondrial fission by the Parkinson disease-associated protein alpha-synuclein. *J. Biol. Chem.* **286**, 20710–20726. 10.1074/jbc.M110.213538 (2011). 21489994 10.1074/jbc.M110.213538 PMC3121472

[72] Pozo Devoto, V. M. et al. alphaSynuclein control of mitochondrial homeostasis in human-derived neurons is disrupted by mutations associated with Parkinson’s disease. *Sci. Rep.* **7**, 5042. 10.1038/s41598-017-05334-9 (2017). 28698628 10.1038/s41598-017-05334-9 PMC5506004

[73] Guan, Y. et al. Pathogenic mutations differentially regulate cell-to-cell transmission of alpha-synuclein. *Front. Cell Neurosci.* **14**, 159. 10.3389/fncel.2020.00159 (2020). 32595456 10.3389/fncel.2020.00159 PMC7303300

[74] Boyer, D. R. et al. Structures of fibrils formed by alpha-synuclein hereditary disease mutant H50Q reveal new polymorphs. *Nat. Struct. Mol. Biol.* **26**, 1044–1052. 10.1038/s41594-019-0322-y (2019). 31695184 10.1038/s41594-019-0322-y PMC6907165

[75] Bagchi, D. P. et al. Binding of the radioligand SIL23 to alpha-synuclein fibrils in Parkinson disease brain tissue establishes feasibility and screening approaches for developing a Parkinson disease imaging agent. *PLoS ONE* **8**, e55031. 10.1371/journal.pone.0055031 (2013). 23405108 10.1371/journal.pone.0055031 PMC3566091

[76] Leupold, L. et al. The quest for anti-alpha-synuclein antibody specificity-lessons learnt from flow cytometry analysis. *Front. Neurol.* **13**, 869103. 10.3389/fneur.2022.869103 (2022). 35911883 10.3389/fneur.2022.869103 PMC9334871

[77] Roehm, N. W., Rodgers, G. H., Hatfield, S. M. & Glasebrook, A. L. An improved colorimetric assay for cell proliferation and viability utilizing the tetrazolium salt XTT. *J. Immunol. Methods* **142**, 257–265. 10.1016/0022-1759(91)90114-u (1991). 1919029 10.1016/0022-1759(91)90114-u

