# Supplementary material for: Effect of hydrogen sulfide on alpha-synuclein aggregation and cell viability
Source: Sci Rep. 2025 May 4;15:15597. doi: 10.1038/s41598-025-99794-z (PMC12050307; doi:10.1038/s41598-025-99794-z)

**Effect of hydrogen sulfide on alpha-synuclein aggregation and cell viability**

**Elena Ostrakhovitch^1,2^, Eun-Suk Song^1,2^, Johannah E. Stegemann^1,2^, Michael McLeod^1^**, **and Tritia**

**R. Yamasaki^1,2,^** *

**SUPPLEMENTARY FIGURE LEGENDS**

**Suppl Figure 1**. Comparative analysis of in vitro generated wt- and mutated α-Syn fibrils. A-D, Analysis of in vitro α-Syn fibril growth of spontaneous (non-seeded) (A) and (C) self-seeded aggregation. Calculation of spontaneous (B) and PFF self-seeded (D) protein aggregation rate (PAR). PAR was calculated as the as the inverse of lag-phase (1/h). E-I, Spectra of cross-seeded aggregation of α-Syn monomer.

**Suppl Figure 2**. A, Fibril diameter for mutations are plotted against the same wt values for each graph measured from TEM images. B, The ﬁbril diameter distribution of wt and A53T α-Syn seeded with wt and A53T PFF in the absence and the presence of NaHS. The value for each graph were measures from TEM Images using VELOX software.

The ﬁgures are representative of three independent experiments with 3 grids analyzed per condition.

**Suppl Figure 3**. A, Analysis of propagation of internalized wt- and mutated α-Syn PFF in HEK 293T cells overexpressing A53T α-Syn tagged with YFP (HEK 293T wt-YFP) at 24, 48 and 72 hours. B, Analysis of propagation of internalized wt- and mutated α-Syn PFF in HEK 293T cells overexpressing wt α-Syn tagged with YFP (HEK 293T wt-YFP).

Cells were transduced with 10 nM A30P-, H50Q-, G51D-, A53T-, and wt-αSyn seeds for 24 hours and inclusions were validated with fluorescent microscopy (Olympus). A, representative images. B, Analysis of cells with seeds. *p< 0.05, ***p< 0.0005, ****p<0.0001

**Suppl Figure 4**. Representative TEM images. A, wt α-Syn and α-Syn bearing mutation PFF. Scale bars, 100 nm. B, wt α-Syn and α-Syn bearing mutation at A53T ﬁbrils seeded with wt and A53T PFF in the absence and the presence of 1 µM NaHS. Scale bars, 200 nm.

**Suppl Figure 5**. NaHS prolongs the lag phase and ultimately delays the formation of fibrils.

A, Effect of different concentrations of NaHS on ThT fluorescence in the absence of α-Syn protein. B, Comparison of lag time for fibril growth of wt-α-Syn and A53T-α-Syn seeded with α-Syn variants (H50Q, G51D, and A53T) in the absence and presence of 1µM NaHS. C, Analysis of propagation of internalized wt- and mutated α-Syn PFF in HEK 293T cells overexpressing familial point mutation A53T tagged with YFP (HEK 293T A53T-YFP). Cells were transduced with 1 nM α-Syn variants (A30P, H50Q, and G51D) in the absence and presence of 1µM NaHS seeds for 24 hours and inclusions were validated with confocal microscopy (Nikon Eclipse Ti2).

**Suppl Figure 6**. PK sensitivity of wt and A53T α-Syn ﬁbrils produced by seeding without and with PFF under ThT conditions in the absence and presence of 1 µM NaHS. A, Coomassie staining after proteinase K (PK)-digestion of wt α-Syn ﬁbrils formed by seeding with wt-PFF in the absence and presence of 1 µM NaHS and A53T α-Syn ﬁbrils formed by seeding with A53T-PFF in the absence and presence of 1 µM NaHS. B, Western-blot immunostaining after proteinase K (PK)-digestion of wt α-Syn ﬁbrils formed by seeding with wt-PFF in the absence and presence of 1 µM NaHS and A53T α-Syn ﬁbrils formed by seeding with A53T-PFF in the absence and presence of 1 µM NaHS. C, Western-blot immunostaining after proteinase K (PK)-digestion of wt α-Syn and A53T α-Syn ﬁbrils formed in the absence and presence of 1 µM NaHS. D, α-Syn dot-blot immunostaining after proteinase K (PK)-digestion of wt α-Syn ﬁbrils formed by seeding with wt-PFF in the absence and presence of 1 µM NaHS. E, α-Syn dot-blot immunostaining after proteinase K (PK)-digestion of A53T α-Syn ﬁbrils formed by seeding with A53T-PFF in the absence and presence of 1 µM NaHS.

The ﬁgures are representative of three independent experiments with 3 grids analyzed per condition.

**Suppl Figure 7**. Slow-releasing donor of H2S, GYY4137, diminished the seeding activity but not cytotoxicity of wt-α-Syn inclusions.

A, Representative confocal images of HEK 293T A53T-YFP cells seeded with wt-PFF and treated with GYY4137. The seeding was validated with confocal microscopy (Nikon Eclipse Ti2). The cells were transduced with 1 nM wt-α-Syn seeds for 48 hours. B, Analysis of α-Syn inclusions formed in HEK 293 A53T cells seeded with 1nM wt-PFF in the absence and the presence of 100 µM GYY4137. C, Analysis of cytotoxicity of 10nM α-Syn wt fibrils in the absence and the presence of 100 µM GYY4137. Cell viability was assessed using XTT assay.

Statistical significance of 5 independent experiments is shown as *p≤0.05, ** **p ≤0.0001.

**Suppl Figure 8**. A-C, Full-length PK digestion Coomassie gel images in Fig. 3 G-I. D, Full-length PK digestion Coomassie gel image in Suppl. Fig. 6A. E, Full-length PK digestion WB image in Suppl. Fig. 6B.

Supplementary Figure 1


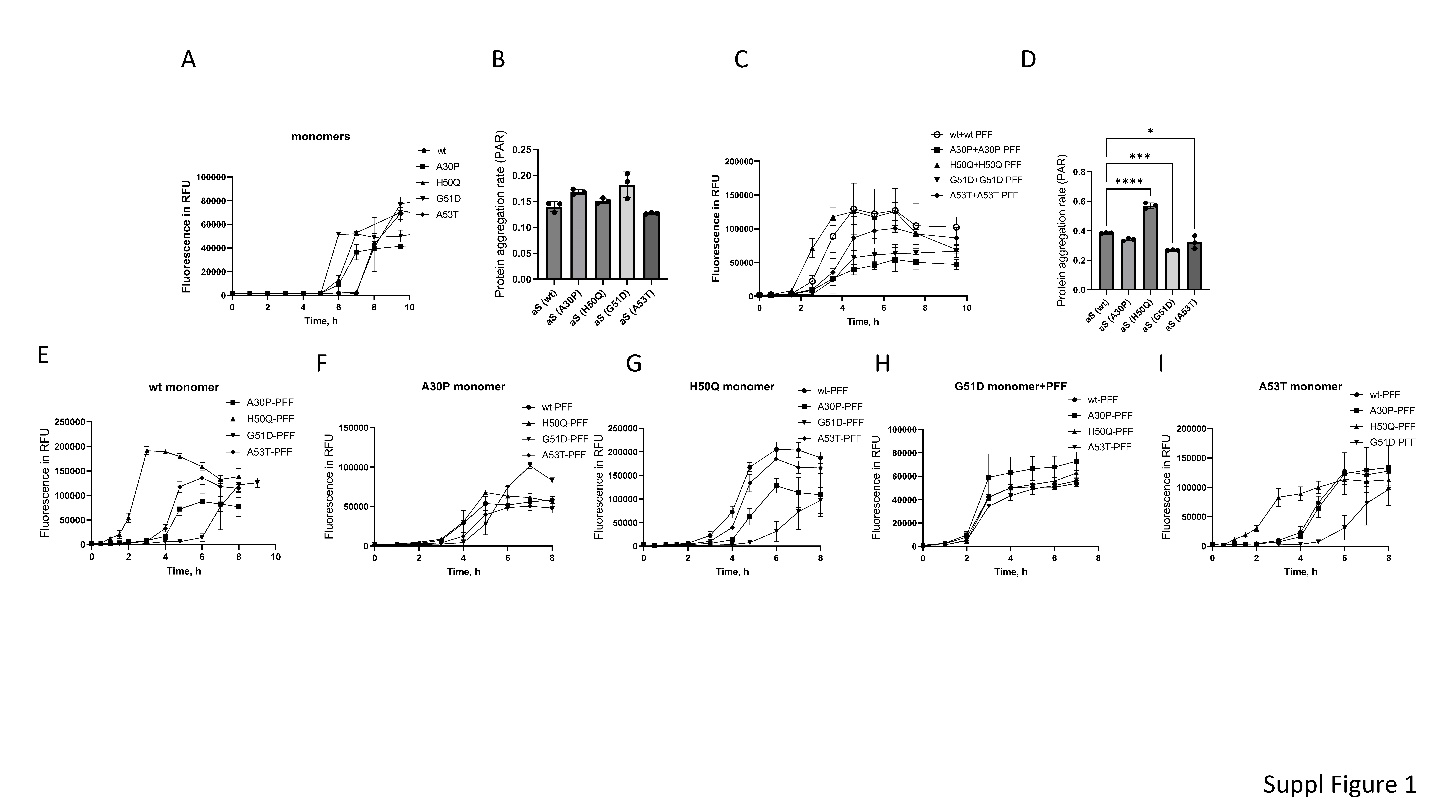


Supplementary Figure 2


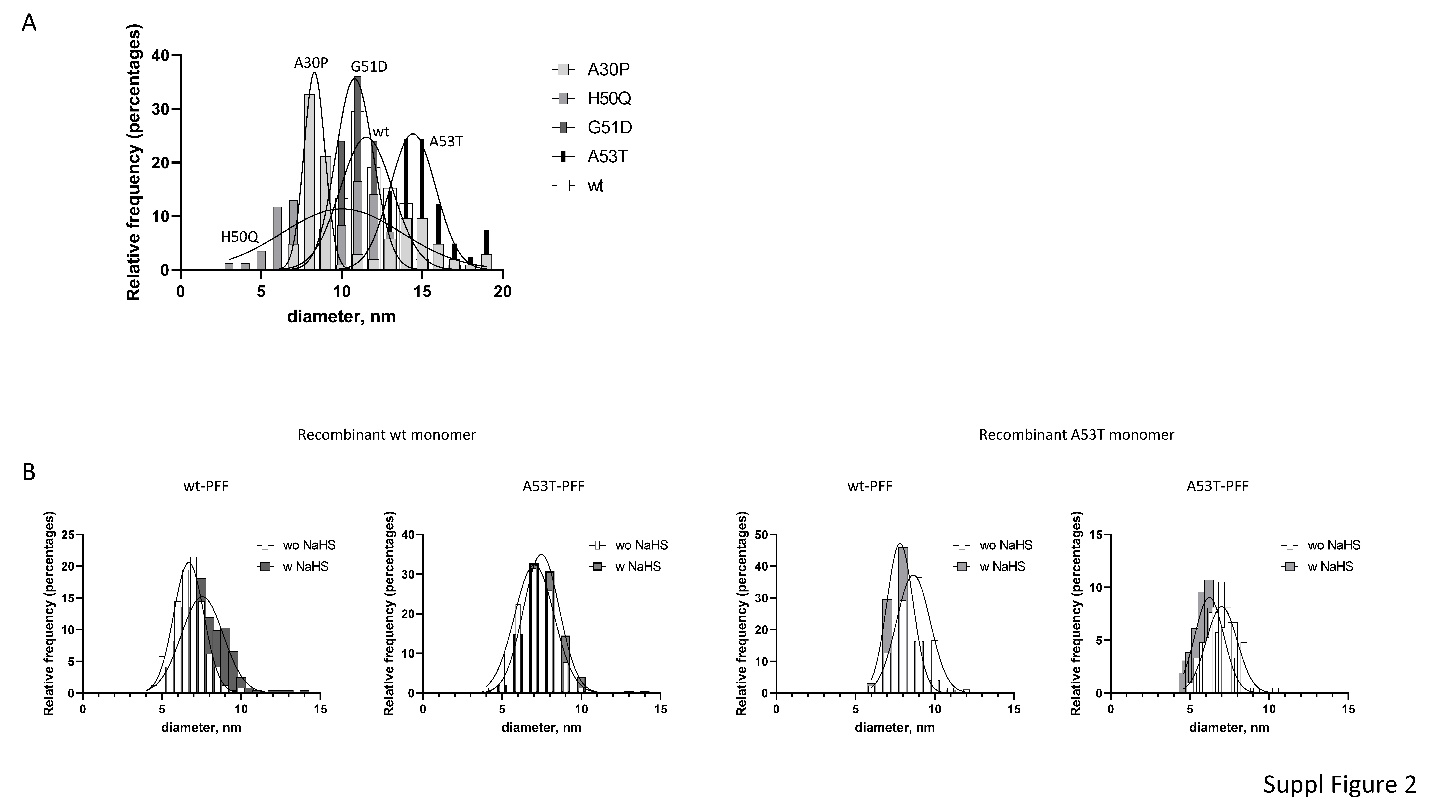


Supplementary Figure 3


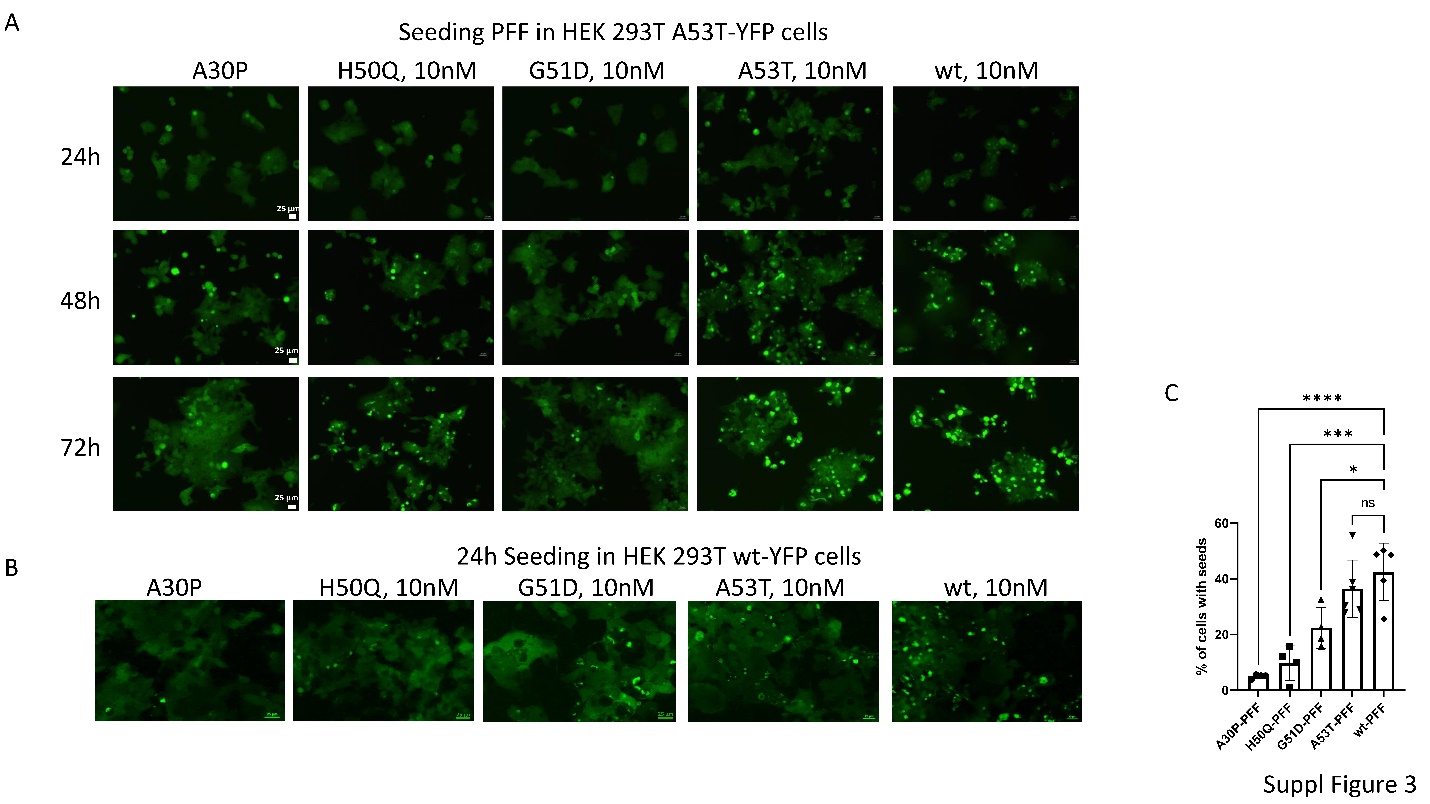


Supplementary Figure 4


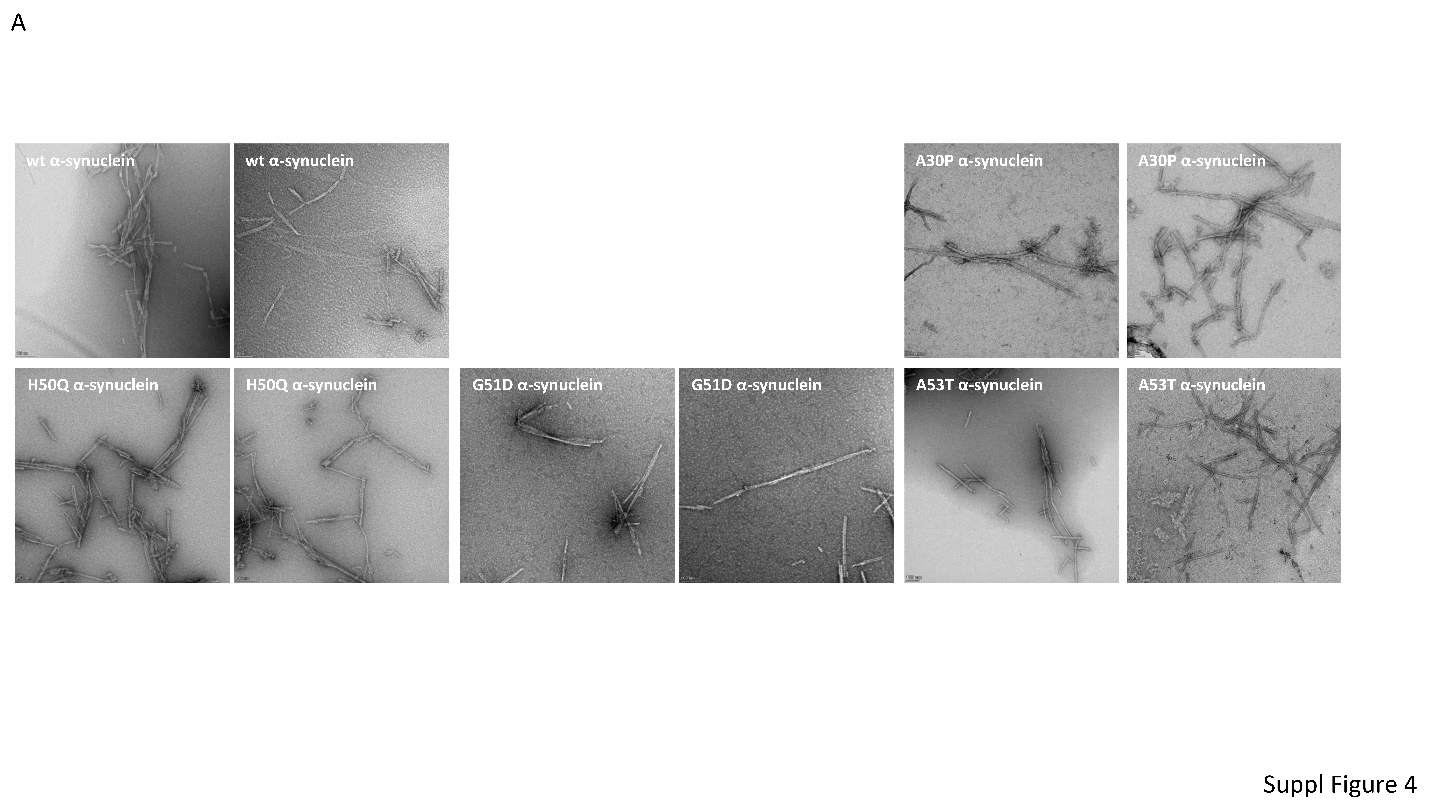


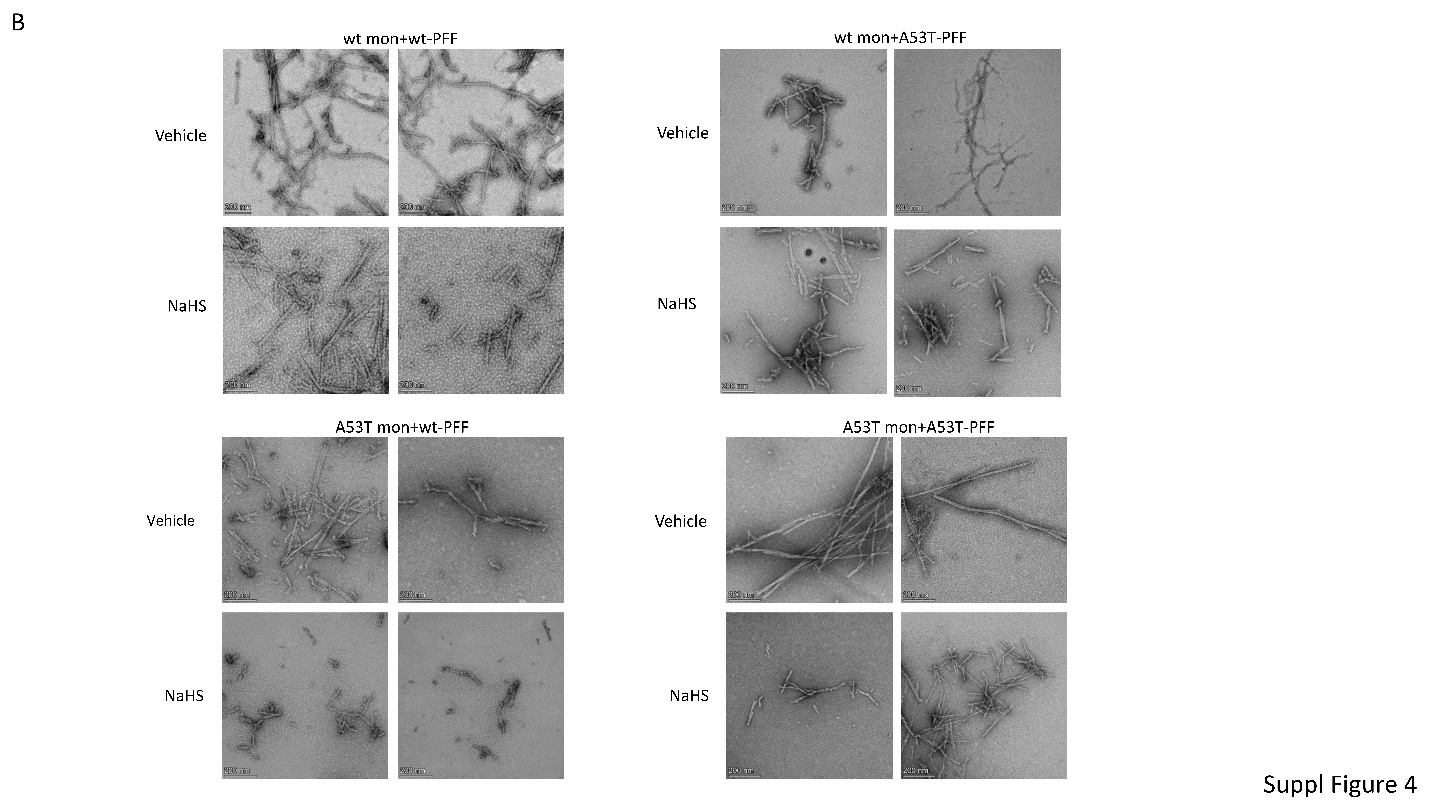


Supplementary Figure 5


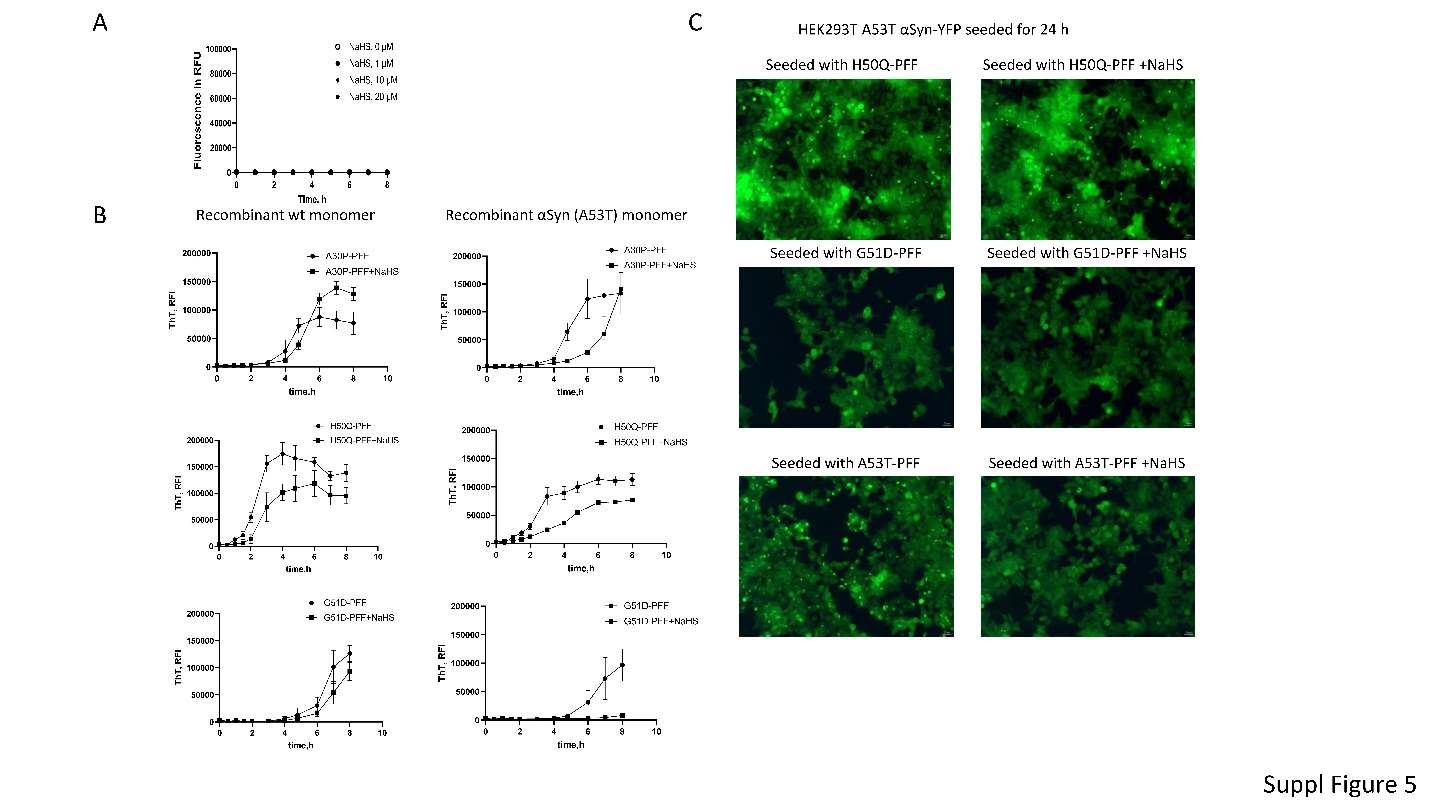


Supplementary Figure 6


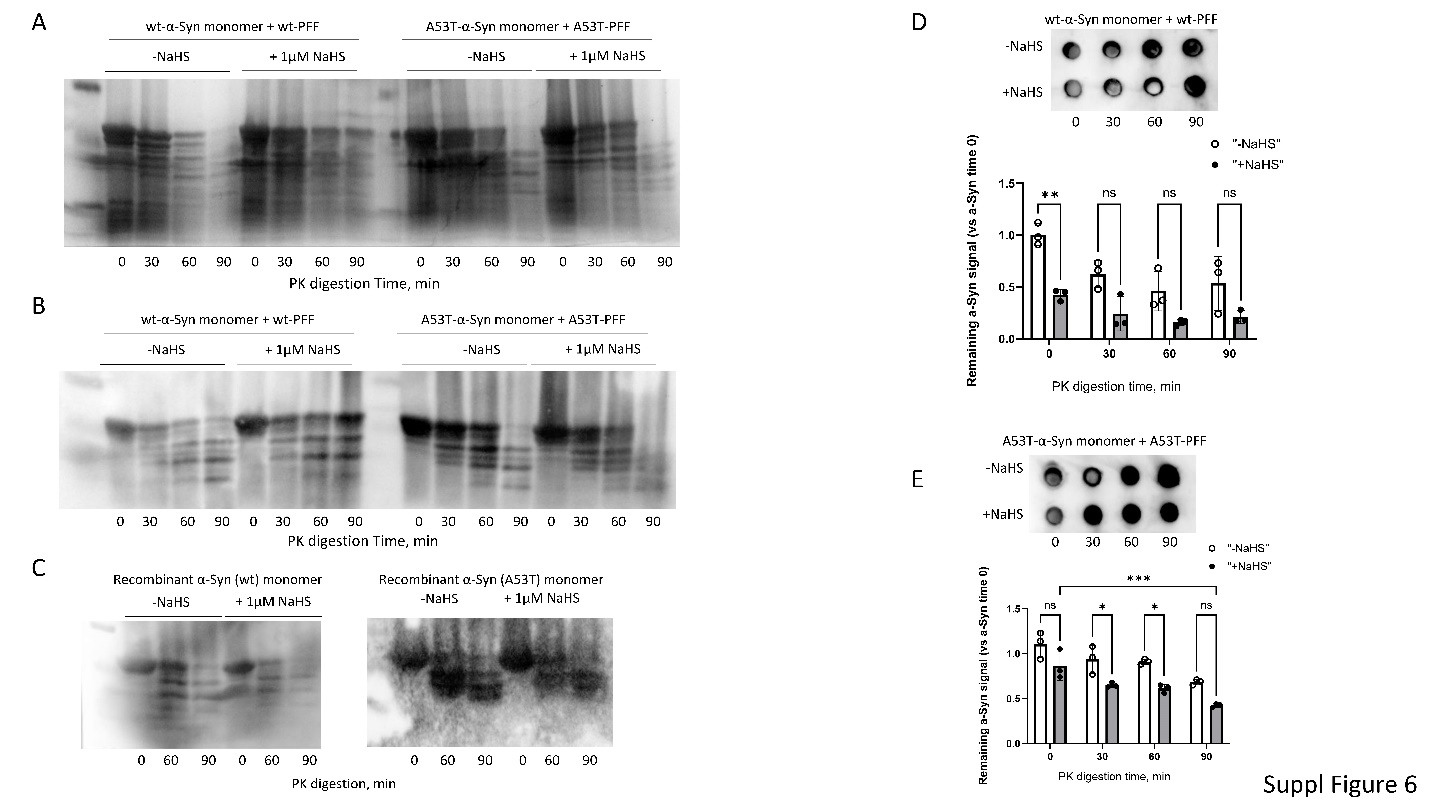


Supplementary Figure 7


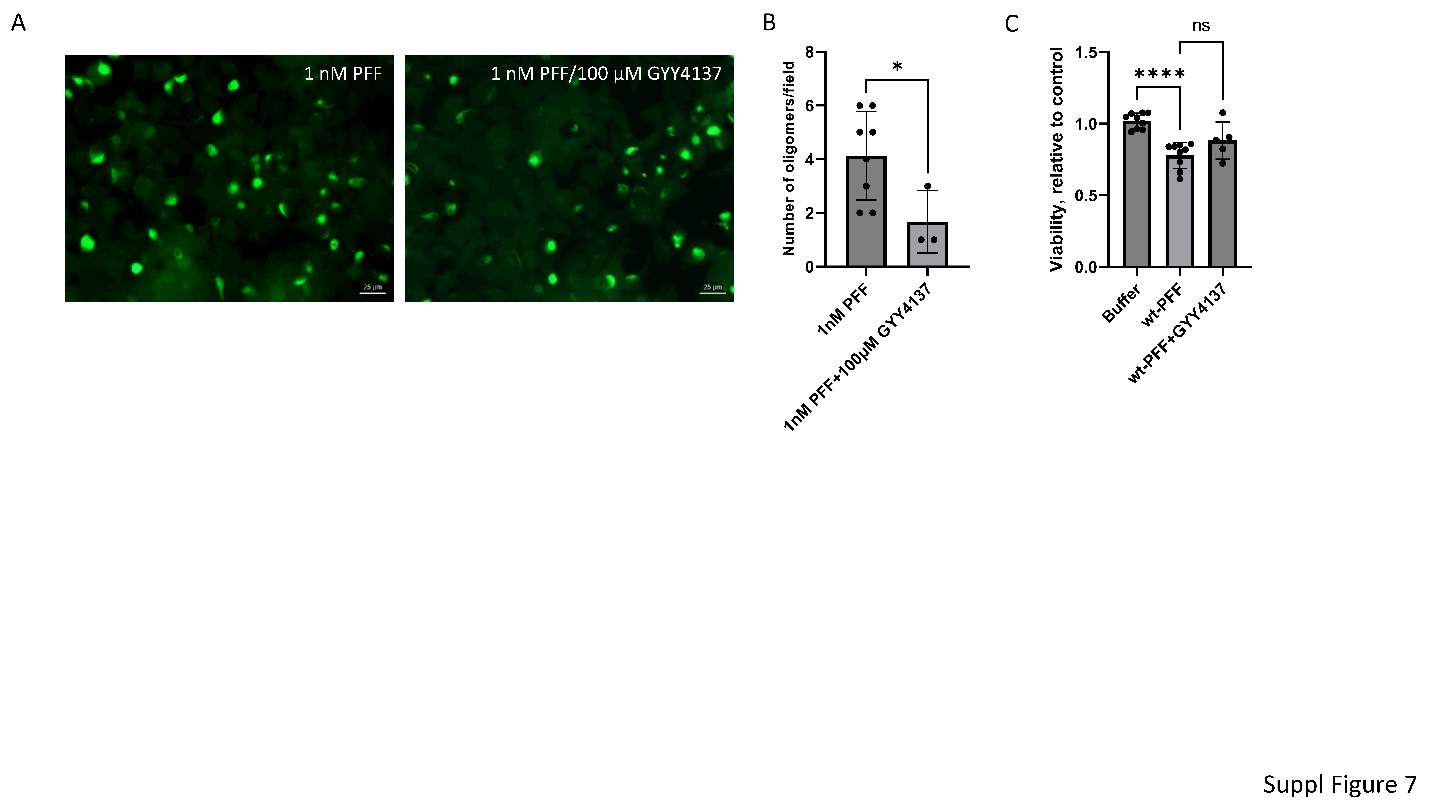


Supplementary Figure 8


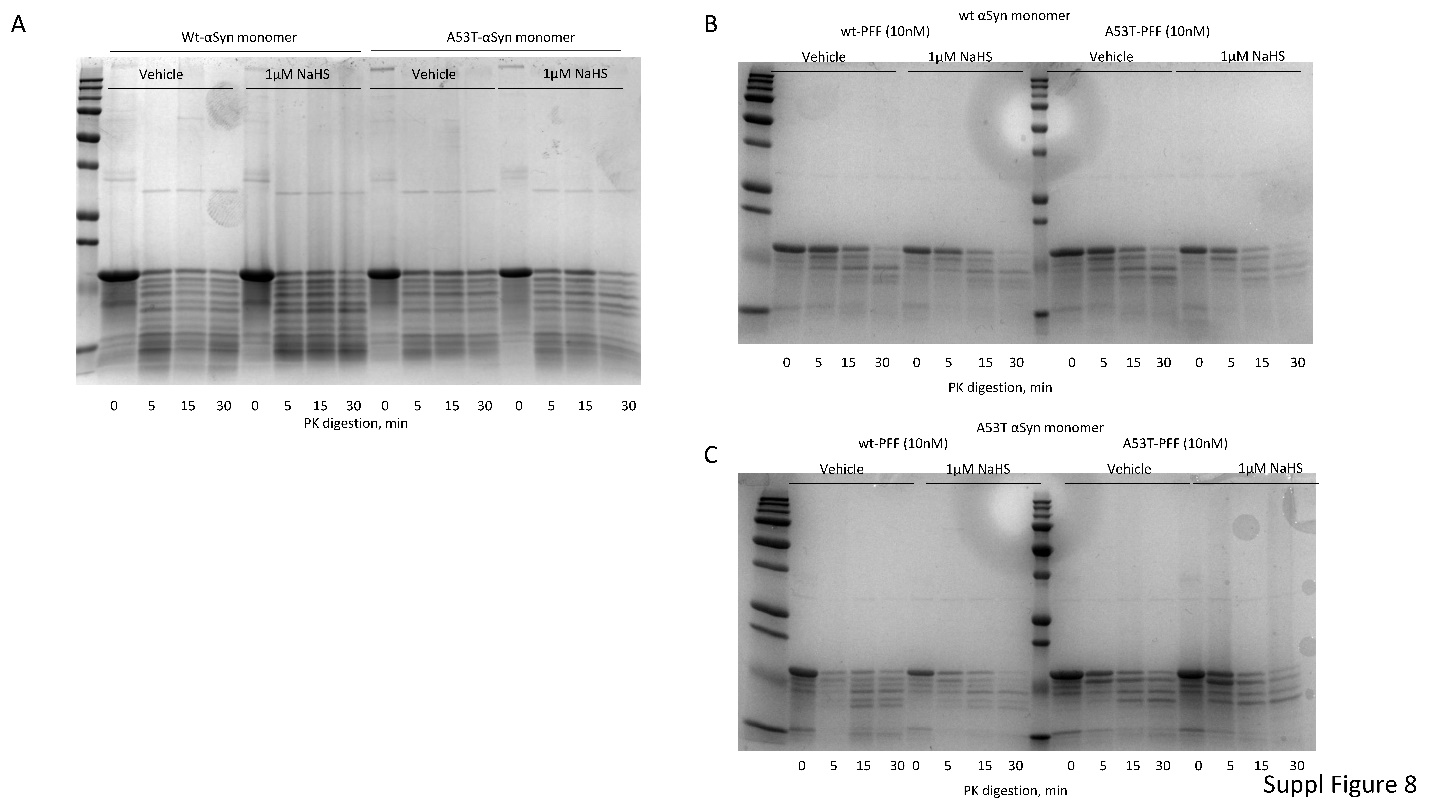


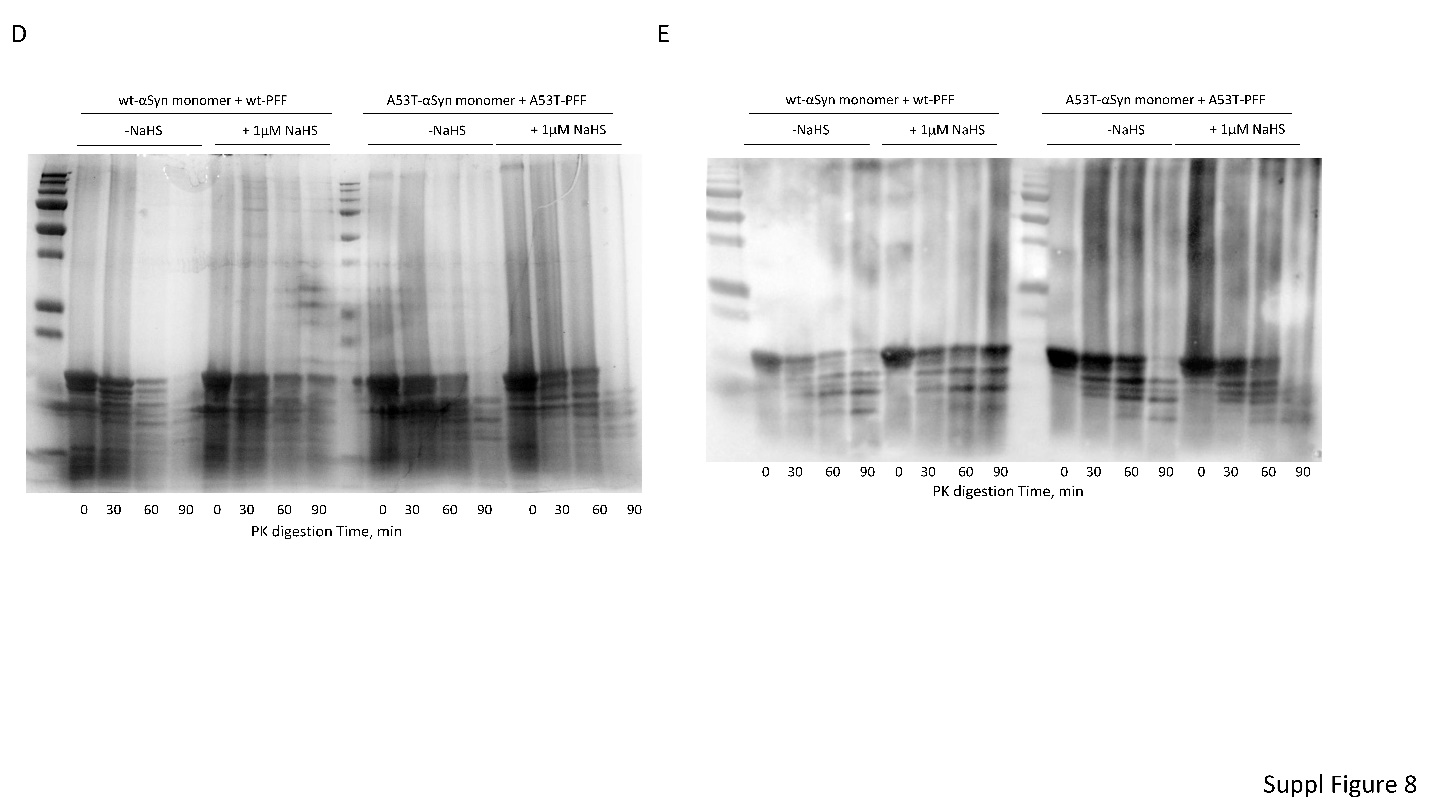

Supplement: Supplementary file 1 — Supplementary Material 1 [file 41598_2025_99794_MOESM1_ESM.docx]
